# Novel prefabricated joints for underground silos with steel cavity and concrete-filled cavity: Axial compression tests and numerical simulations

**DOI:** 10.1371/journal.pone.0361019

**Published:** 2026-10-23

**Authors:** Jinmi Li, Jun Chuai

**Affiliations:** 1 School of Architecture and Civil Engineering, Zhengzhou University of Industrial Technology, Zhengzhou, China; 2 School of Architectural Engineering, Kunming University of Science and Technology, Kunming, China; Tongji University, CHINA

## Abstract

In terms of new materials, innovative technologies, and advanced construction techniques, the prefabricated steel plate-concrete composite underground silo offers an effective solution to the key challenges of cost, construction duration, and waterproofing associated with conventional reinforced concrete underground silos. However, joints represent critical weak components in prefabricated structures, and the development of simple and efficient joint configurations is essential for the further advancement and application of novel prefabricated underground silos. To address this issue, two novel prefabricated joints, namely the steel cavity joint and the concrete-filled cavity joint, are proposed, and their axial compressive performance is investigated. First, full-scale axial compression tests are conducted to evaluate the load-bearing, deformation, yielding, and failure behavior of specimens incorporating different joint configurations. Subsequently, nonlinear finite element analysis of both specimens is performed using ABAQUS to further elucidate their load-transfer mechanisms. Furthermore, the effects of key joint parameters on the axial compressive performance are discussed. The results show that the specimen with a steel cavity joint primarily fails due to the instability of external waterproof steel plate under axial compression, with the steel plate contributing approximately 41% of the applied load during the elastic stage. In contrast, the specimen with a concrete-filled cavity exhibits crushing failure of prefabricated concrete blocks, with the cavity concrete contributing approximately 75% of the applied load during the elastic stage. Compared with the steel cavity joint, the concrete-filled cavity joint exhibits significantly improved initial stiffness and achieves an increase of more than 28% in peak load capacity. Moreover, the concrete strength grade in the cavity can be appropriately reduced to optimize material utilization efficiency.

## Introduction

Food security has always been a major strategic issue related to national economic development, social stability, and national autonomy. As an essential facility for grain storage and distribution, the granary plays an irreplaceable role in ensuring the security of grain supply. In the context of the dual carbon strategy and the pursuit of high-quality development, land conservation, environmental sustainability, and low-carbon performance in granary construction has become a major challenge for the grain industry.

Compared with traditional aboveground granaries, underground granaries offer significant advantages, including lower storage temperatures, environmental friendliness, and land conservation [1–3]. Previous studies have investigated the structural performance [4–6] and design methods of reinforced concrete underground silos. These studies have consistently indicated that reinforced concrete remains the predominant structural material for underground silos [7, 8].

However, due to its relatively high costs and long construction periods, as well as deficiencies in waterproofing performance and buoyancy resistance, such underground granaries are difficult to promote and apply on a large scale. Therefore, numerous researchers have carried out extensive studies and proposed a variety of improvement strategies. Lining the inner surface of concrete silo walls with plastic panels represents a relatively novel approach [9–11]. In this system, polypropylene (PP) plastic panels are integrally cast with concrete through plastic joints [12,13]. Zhang et al. [9] investigated the stress distribution and mechanical behavior of the plastic waterproof liner under water pressure through hydrostatic tests and finite element analysis. They further examined the relationship between joint spacing and the water pressure resistance of the structure. Wang et al. [12] studied the pull-out performance of smooth and grooved PP rods embedded in concrete. The results revealed that the bond-slip curve between PP rods and concrete can be divided into three stages: an elastic stage, a local cracking stage, and a descending stage, with the surface morphology ofPP rods having the greatest influence on their pull-out performance. Despite these efforts, the relatively limited development of existing research systems and the lack of relevant codes and standards have restricted the practical application of these research outcomes. At present, the findings remain primarily serve as reference for the waterproofing and anti-floating design of underground silos, and the long-standing challenges associated with insufficient waterproofing and anti-floating performance have not yet fully addressed.

Furthermore, Wang et al. [14] proposed a prefabricated steel-concrete underground grain silo (referred to as the prefabricated underground silo) system. This innovative system integrates prefabrication with composite structural technology [15] and utilizes a top-down construction method to avoid extensive excavation, providing an effective solution to the challenges of foundation pit support. The composite structure is lined with single-sided steel plates that function not only as structural components but also as waterproofing layers [16]. This configuration largely overcomes the limitations associated with large-diameter reinforced-concrete underground silos and has been progressively applied in both aboveground and underground construction projects, including bridges, tunnels, and foundation pits, demonstrating favorable economic and social benefits [17–19].

Joints are the most critical and vulnerable components in prefabricated structures, with their connection methods and mechanical properties serving as key factors influencing the overall structural strength and stability [20–23]. In prefabricated structures for tunnels and metro projects, extensive research has been conducted on joint types and their mechanical behavior [22–26]. For example, Zhou et al. [27] investigated the dynamic response and damage characteristics of gravity-type mortise-and-tenon joints, bolted mortise-and-tenon joints, and steel-plate-reinforced mortise-and-tenon joints under impact loading. Their findings highlighted the necessity of enhancing joint stiffness in the impact-resistant design of prefabricated tunnel structures. Zhang et al. [28] carried out shear model tests under four working conditions to examine the performance of socket joints in utility tunnels, and conducted a comparative analysis of failure modes and shear resistance. Jia et al. [29] conducted full-scale tests on shield tunnel segment joints to investigate their flexural behavior and elucidate the failure process and load-bearing mechanism of joints. Zhang et al. [30] combined full-ring tests with numerical simulations to explore the mechanical behavior and load-bearing mechanism of shield tunnel linings with socket joints. Furthermore, Chen et al. [31] conducted physical and numerical studies on the seismic performance characteristics of multi-story and multi-span prefabricated subway station structures. The results indicated that the column ends in the upper layer are the weak points of the multi-story prefabricated subway station structure, experiencing significant damage during earthquakes. Zhao et al. [32] proposed a resilient slip-friction connection (RSFC-SCC) for prefabricated underground structures to enhance their seismic performance and facilitate rapid post-earthquake recovery. Using a subway as a case study, they conducted nonlinear time history analyses to validate the effectiveness and elastic characteristics of the RSFC-SCC. By contrast, research on joints in prefabricated underground silos remains relatively limited because this structural system is still at an early stage of development. Wang et al. [33–35] proposed a novel dry joint composed of three steel plates, and through full-scale comparative experiments, evaluated the flexural and compressive performance of specimens with and without joints. Building on this, Zhang et al. [36–38] further introduced two joint forms, embedded trapezoidal steel plate joints and bolt-weld hybrid joints, and studied their failure modes and flexural capacity through symmetric bending tests and finite element simulations. The results demonstrated that these joint forms exhibited flexural and compressive performance comparable to that of jointless specimens. Nevertheless, the three-steel-plate dry joint requires numerous steel plates and welds, while the embedded trapezoidal steel plate joint and the bolted-welded hybrid joint are constrained by construction difficulties and complex structural detailing, respectively. These drawbacks limit their practicality and hinder their widespread application in engineering practice.

To develop an efficient, economical, and easy-to-construct connection for prefabricated steel-concrete composite underground grain silo walls, this study proposes two novel joint configurations based on the three-steel-plate dry joint reported in Ref [34]. Compared with existing underground silo joints, the proposed configurations provide a simpler structural layout, fewer welded connections, and improved constructability. Full-scale axial compression tests and numerical simulations were conducted on silo wall specimens with different joint configurations to evaluate their mechanical performance and reveal their load-transfer mechanisms. Through a comparative analysis of the mechanical behavior of the proposed joint types, feasible vertical connection schemes for silo walls under different service conditions were identified. The findings provide valuable insights into the design of vertical wall joints in prefabricated underground silos and contribute to the further development and engineering application of prefabricated underground grain storage systems.

## Axial compression test

A large-diameter reinforced concrete underground grain silo (test silo) constructed in Henan Province was selected as the structural prototype. The silo was designed with a storage capacity of approximately 5,000 t (wheat), an internal diameter of 25 m, and a composite wall approximately 20 m in height and 0.31 m in thickness. The structural scheme is described in detail in the literature [14], and its cross-sectional schematic is shown in Fig 1a. The wall structure of the prefabricated underground granary is illustrated in Fig 1b. The horizontal joint between the upper and lower prefabricated blocks along the circumferential direction of the silo wall is defined as the circumferential joint, and the vertical joint between the left and right prefabricated blocks along the height of the silo wall is defined as the vertical joint. According to the column-shell theory, it can be seen that the silo wall is mainly circumferentially compressed under the action of external water and soil pressure. Therefore, this study focuses on analyzing the axial compressive behavior of the vertical joints in the composite silo wall.

**Fig 1:**
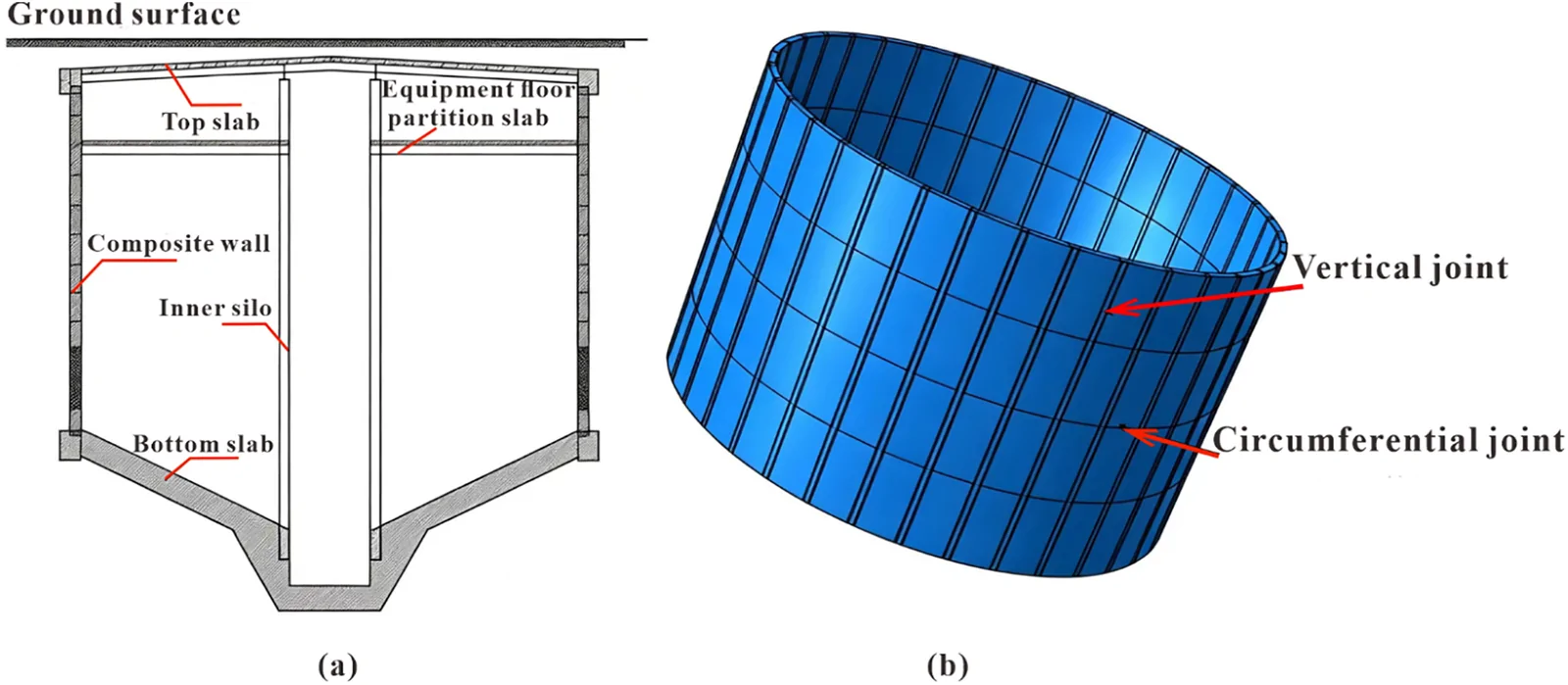
Structural configuration of the prefabricated underground granary. (a) cross-sectional view of the prefabricated underground granary. (b) schematic of the prefabricated underground granary wall structure.

### Design of specimens and joints

Based on the structural configuration shown in Fig 1 and the provisions of the Code for Design of Composite Structures [39], the prefabricated composite silo wall segments and their corresponding joint configurations were designed. Although the prototype wall is curved, curved specimens require specialized loading equipment and make it difficult to satisfy the desired boundary conditions. Therefore, to ensure that the stress state remained representative of the prototype while facilitating laboratory testing, flat specimens were adopted in place of the curved wall segments. A total of four prefabricated wall segments were fabricated. Two segments were connected through a single joint to form one test specimen, resulting in two full-scale specimens. Both specimens had identical geometric dimensions, with a total length of 2200 mm, a width of 1000 mm, and a thickness of 310 mm. The inner layer of prefabricated blocks consisted of a 10 mm thick steel plate, while the outer layer was composed of 300 mm thick concrete. The inner steel plate was connected to the precast concrete block by means of headed studs. The steel grade was Q355, and the concrete strength grade was C40. The geometric dimension and the three-dimensional configuration of the specimen are shown in Fig 2.

**Fig 2:**
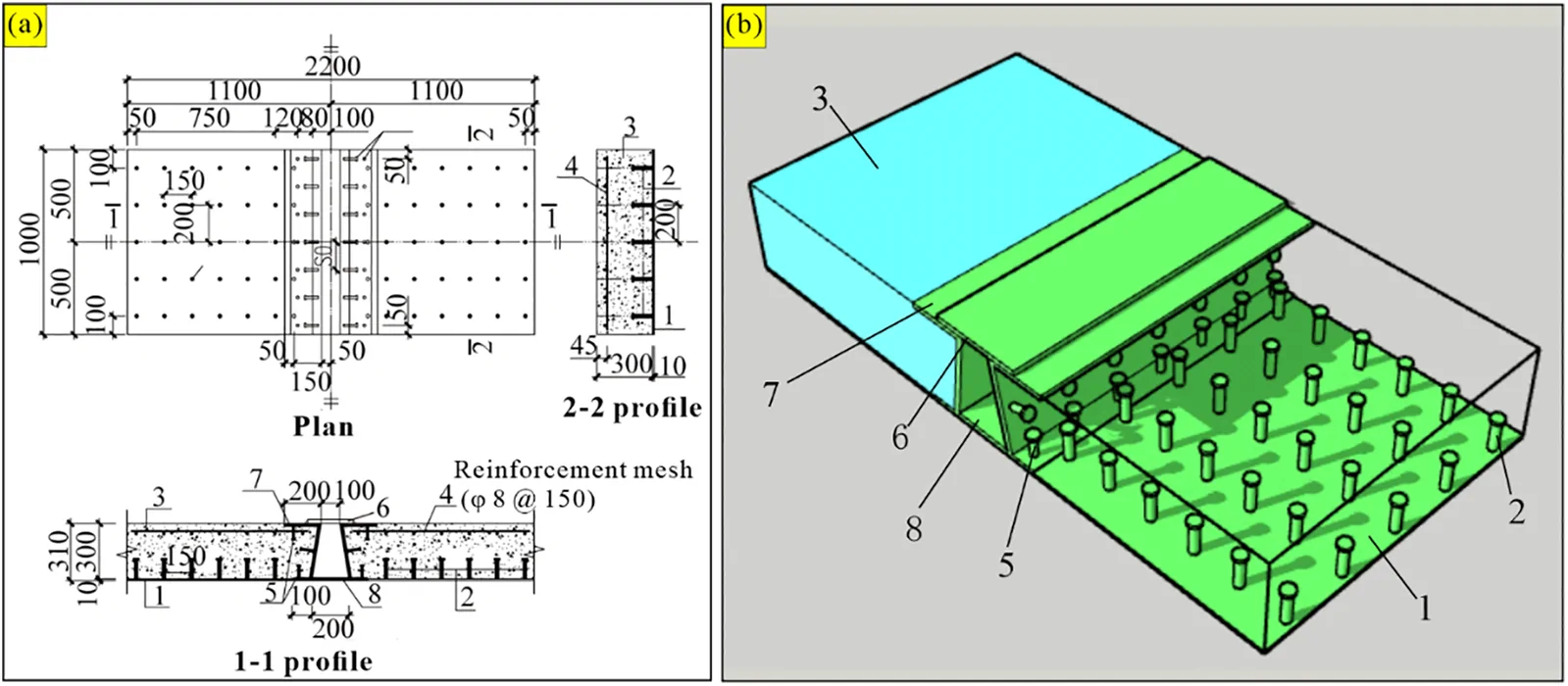
Schematic illustration of the specimen: (a) specimen dimensions (Unit: mm). (b) three-dimensional structural configuration. 1. Inner steel plate, 2. Headed studs (φ 19), 3. Prefabricated concrete block, 4. Reinforcement mesh (φ 8 @ 150), 5. Headed studs (φ 13), 6. External waterproof steel plate, 7. Outside bound steel plate, 8. Internal waterproof steel plate.

Based on the construction characteristics of underground silos, two types of joints were designed, namely the steel cavity (SC) joint and the concrete-filled cavity (CFC) joint. The SC joint is a dry-type connection consisting of two bound steel plates, an external waterproof steel plate, and an internal waterproof steel plate. The bound steel plates were designed with an inclined configuration to facilitate top-down construction and the integrated implementation of foundation pit support [34]. All three components have an equal thickness of 16 mm and are connected together by welds. The external and internal waterproof steel plates are 240 mm and 200 mm long, respectively. The CFC joint adopts a combined dry-wet connection method. Based on the configuration of the SC joint, the cavity is filled with C25 micro-expansive concrete. In this study, the prefabricated steel-plate concrete specimens assembled with the SC joint and the CFC joint are referred to as PSC-SC and PSC-CFC, respectively. The schematic diagram of the structure of the two joints is shown in Fig 3.

**Fig 3:**
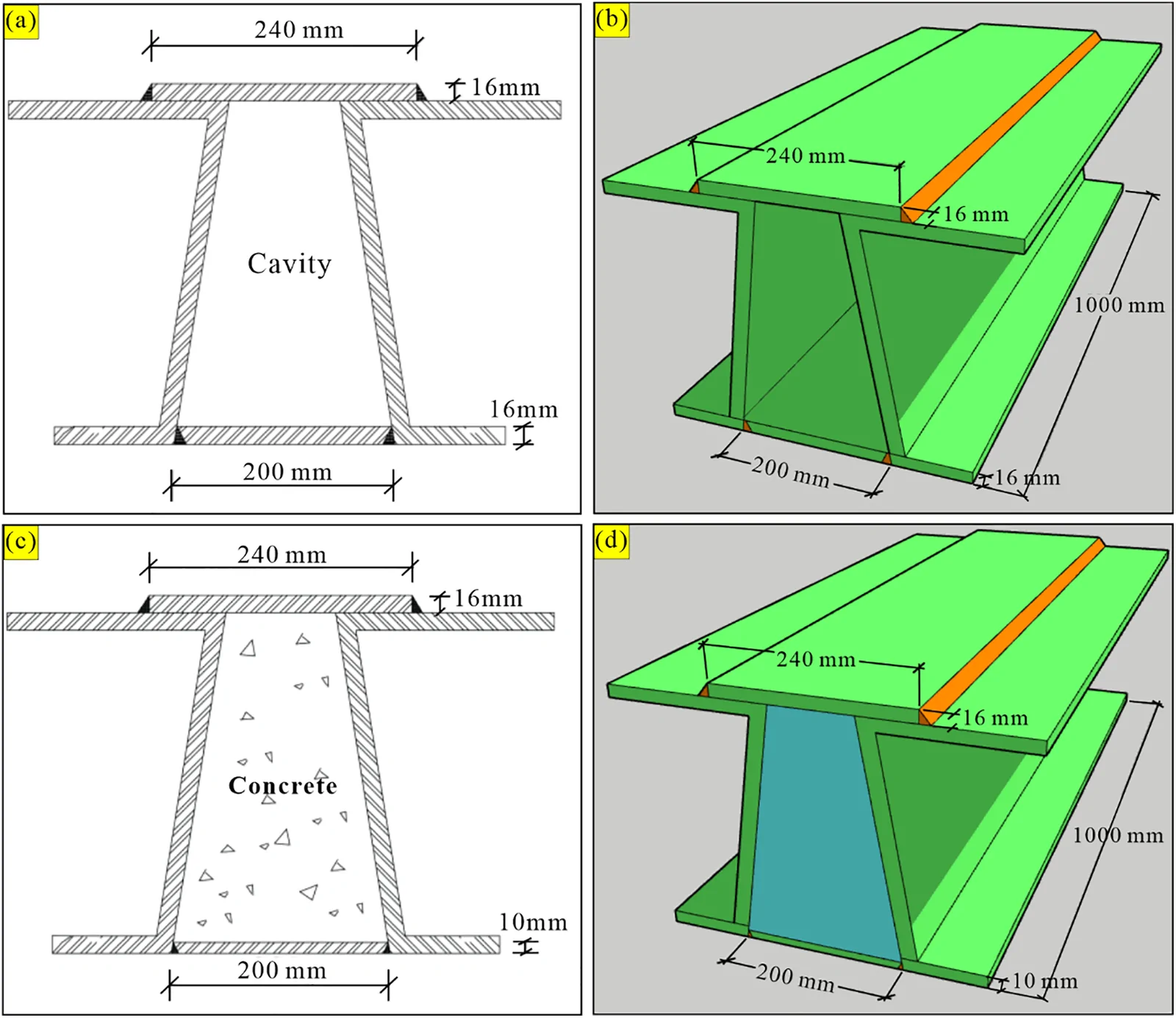
Schematic diagram of SC and CFC joints. (a) Cross-section of SC joint. (b) 3D view of SC joint. (c) Cross-section of CFC joint. (d) 3D view of CFC joint.

### Specimen fabrication and material property testing

As shown in Fig 4 a and Fig 4b, the specimen fabrication process involves the processing of steel components, the pouring and curing of concrete, the joint treatment and the overall assembly of prefabricated blocks. The steel components were fabricated in batches by a structural steel manufacturer in accordance with standardized procedures. The prefabricated concrete blocks were cast using commercially available C40 concrete in batches. Adequate compaction was achieved using a vibrating rod, and the top surface was manually leveled upon completion of the casting. After 28 days of standard curing and joint treatment, the prefabricated blocks were assembled.

**Fig 4:**
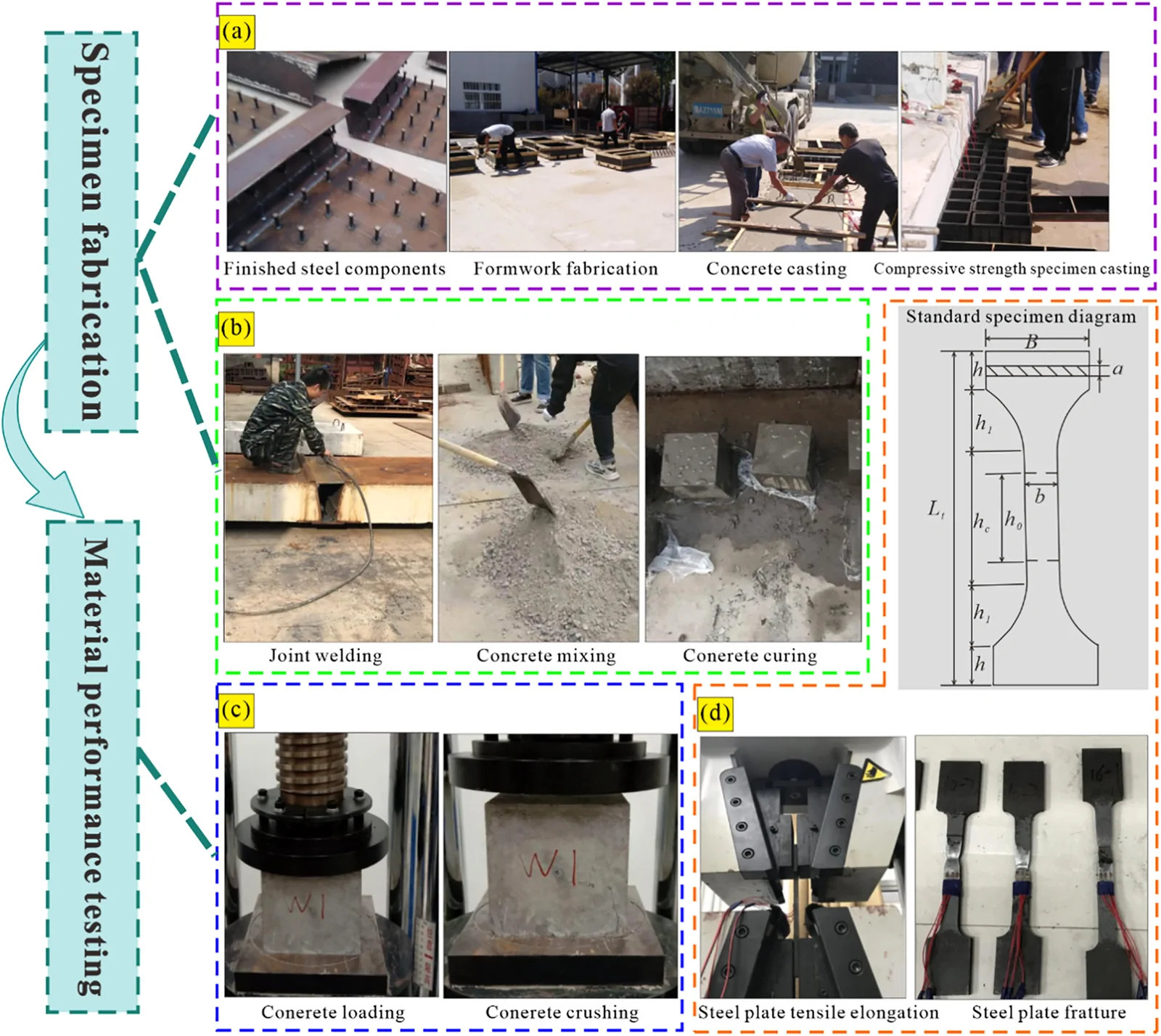
Specimen fabrication and material property testing. (a) Fabrication of prefabricated blocks, including steel component fabrication, formwork assembly, concrete pouring, and curing. (b) Joint treatment process, including joint welding, concrete mixing, and curing, together with the standard specimen geometry. (c) Concrete material property testing, including compressive specimen preparation and loading tests. (d) Steel plate material property testing, including tensile elongation and fracture performance evaluation.

As shown in Fig 4d, standard steel specimens fabricated from the same material as the test specimens were subjected to tensile tests using a universal testing machine. The testing machine has a maximum load capacity of 600 kN and a load measurement resolution of 1/20,000. The measured mechanical properties of steel plates are presented in Table 1. Additionally, standard concrete compressive strength cubes with dimensions of 150 × 150 × 150 mm were cast in accordance with the *Code for Design of Concrete Structures* (GB 50010) [40]. The compression tests were conducted using an automatic compression testing machine (JYE-2000A), as illustrated in Fig 4c, and the resulting concrete strength values are summarized in Table 2.

**Table 1:** Standard dimensions and material properties of steel plates.

Cases	*a* (mm)	*b* (mm)	*r* (mm)	*L*_*0*_ (mm)	*L*_*c*_ (mm)	*L*_*t*_ (mm)	*B* (mm)	*h*_*1*_ (mm)	*H* (mm)	Yield strength *f*_y_ (MPa)	Elastic modulus *E*(MPa)
1	10	20	15	79	105	265	50	15	65	330	2.06 × 10^5^
2	16	20	15	102	130	290	50	15	65	343	2.06 × 10^5^
3	20	20	15	125	150	310	50	15	65	352	2.06 × 10^5^

**Table 2:** Measured concrete strength.

Concrete strength grade	Concrete strength (MPa)	Average measured strength (MPa)
C25	23.4	24.0
C25	24.3	
C25	24.2	
C40	40.7	42.0
C40	43.0	
C40	42.2	

### Experimental method

The loading configuration for the axial compression test is shown in Fig 5. The loading device comprised an electro-hydraulic servo loading system (YAJ-12000) with a maximum loading capacity of 1200 t. As shown in Fig 5b, a restraint was installed to prevent misalignment of the specimen during loading. The data acquisition system adopted the DH3816N data logger, with each unit providing 60 channels. Displacement measurements were conducted using the YHD-50 displacement gauge, which had a measurement accuracy of 0.01 mm. Steel and concrete strain gauges were installed on the corresponding materials to measure strain.

**Fig 5:**
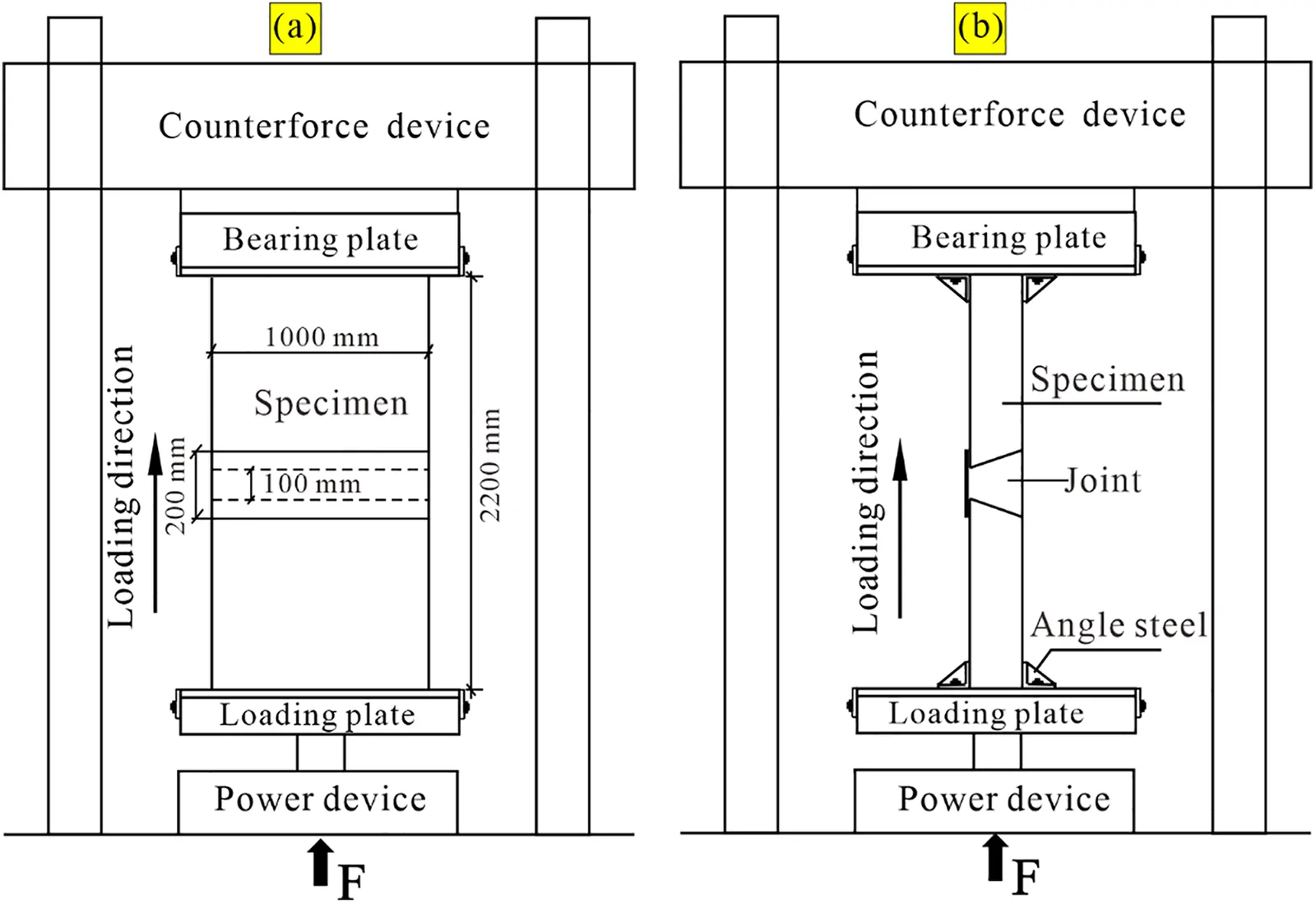
Schematic diagram of the experimental loading device. (a) Front view of the long-column loading system. (b) Side view of the boundary-limiting device.

To investigate the strength and stiffness of silo wall joints under axial pressure, displacement and strain measurement points were arranged on both sides of specimens. As illustrated in Fig 6, PSC-SC and PSC-CFC had an identical layout of displacement and strain gauges. The total axial deformation of the specimen was obtained by subtracting the average displacement of gauges on the top bearing plate (YW9-YW10, YW19-YW20) from the average displacement of those on the bottom loading plate (YW1-YW2, YW11-YW12).

**Fig 6:**
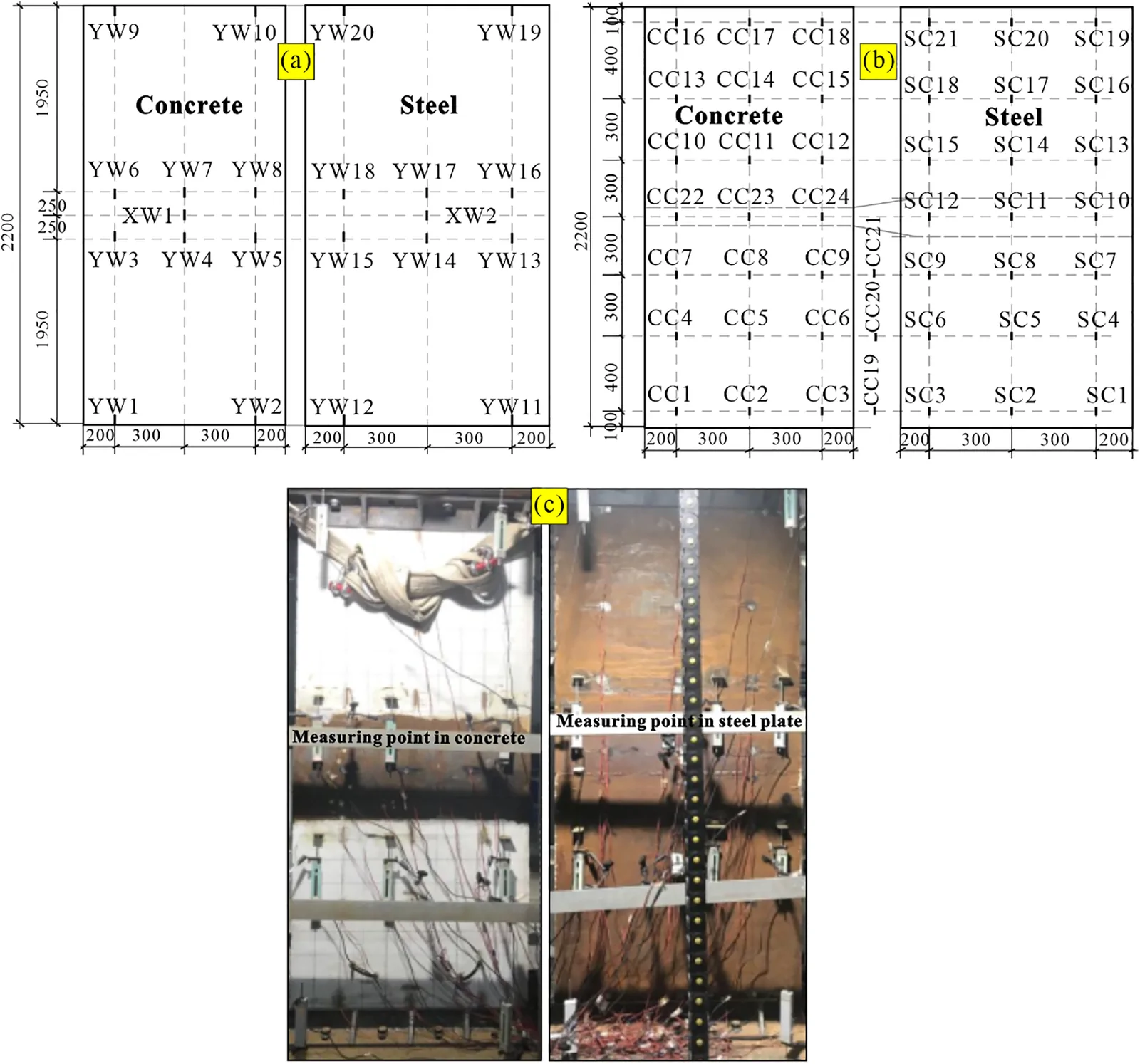
Layout of measurement points. (a) Layout of displacement measurement points, where YW1-YW20 represent axial displacement measurement points and XW1-XW2 represent lateral deflection measurement points. (b) Layout of strain measurement points, including CC1-CC21 for concrete strain gauges and SC1-SC24 for steel strain gauges. (c) Layout of on-site measurement points, showing installation positions of displacement and strain measurement devices on concrete and steel plate surfaces during testing.

According to the *Standard for Test Methods of Concrete Structures* (GB/T 50152) [41], the loading process was divided into two stages: pre-loading and formal loading. During the pre-loading stage, the applied load was limited to no more than 15% of the specimen's predicted bearing capacity determined by finite element analysis. This stage aimed to ensure the proper functioning of the loading system and measurement devices, as well as to ensure reliable contact at the support and loading interfaces. In the formal loading stage, a load-controlled approach was adopted, and each loading increment was set to 10% of the predicted ultimate bearing capacity. Upon reaching 70% of this predicted capacity, the loading method was switched to displacement control, with a displacement increment of 0.5 mm per step and a loading rate of 0.002 mm/s. The loading process continued until either significant yielding occurred at the joints or the specimen’s bearing capacity dropped to 85% of the predicted ultimate capacity. Each loading level was held for 5 minutes to ensure data stability.

## Experimental results and analysis

### Axial force-displacement relationship of PSC-SC and PSC-CFC

(1) Axial load-displacement relationship of PSC-SC

The displacement curve of the PSC-SC under axial pressure and the failure mode are shown in Fig 7. It is evident that the failure is primarily governed by the damage of the external waterproof steel plate at the joint, and the whole compression deformation process of PSC-SC can be divided into three stages: the elastic stage (O_1_-A_1_), the elasto-plastic stage (A_1_-B_1_), and the buckling stage (B_1_-C_1_). The deformation characteristics of each stage are investigated as follows.

**Fig 7:**
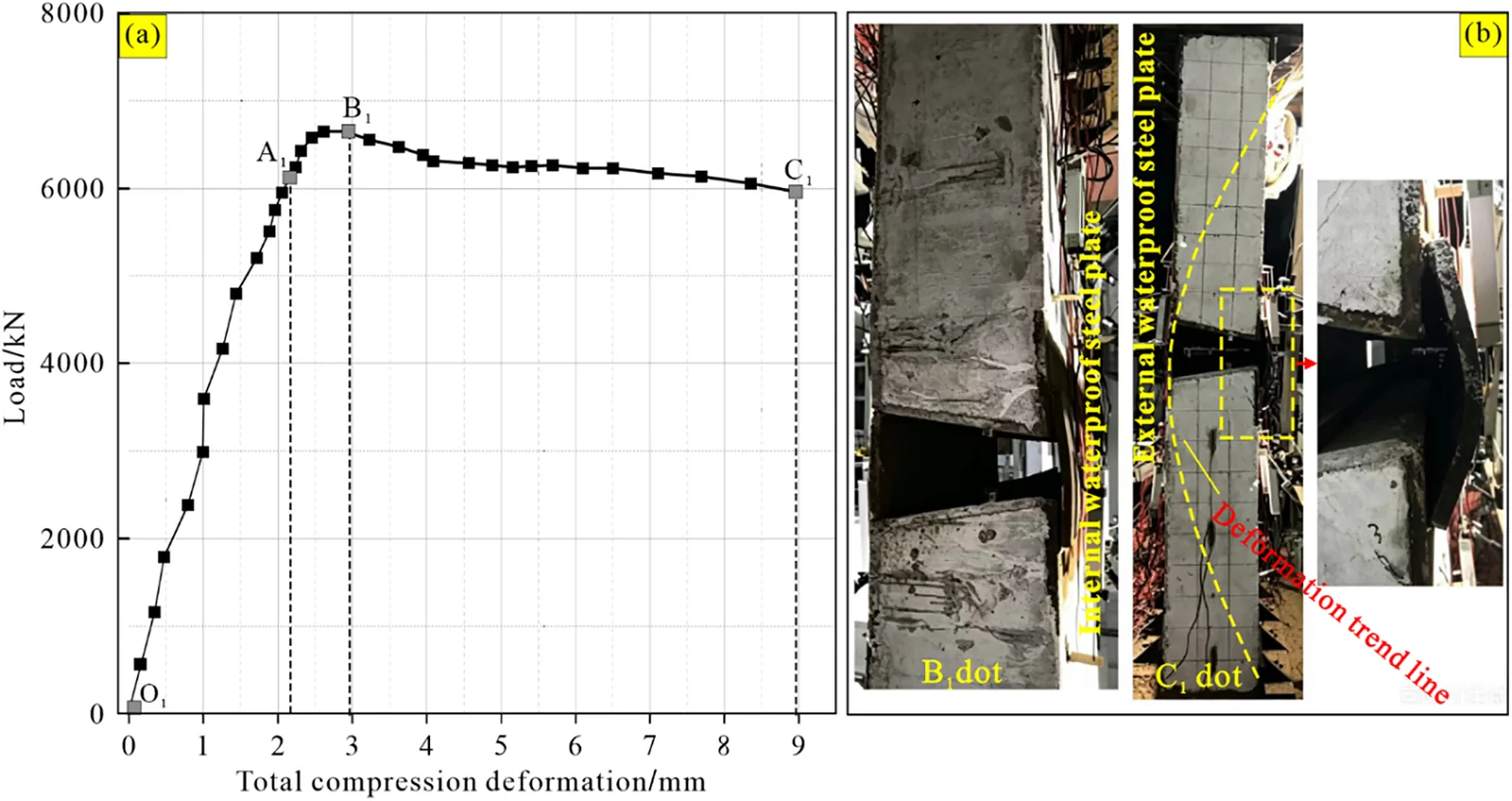
Compressive deformation curve and loading process of PSC-SC. (a) Load-deformation curve under axial compression, illustrating the relationship between applied load and total compressive deformation and identifying key characteristic points (O_1_, A_1_, B_1_, C_1_). (b) Loading process and failure development of the specimen, including internal and external waterproof steel plates and deformation trend visualization during loading.

Elastic stage (O_1_-A_1_): The load-deformation curve of PSC-SC is a straight line with a certain slope, and the compressive stiffness reaches the maximum value in this stage. At the same time, no cracks are observed in the precast concrete blocks, and the connection between the concrete and the inner steel plate via shear studs remains intact. Both the steel plates and welds at the joint remain undamaged, and no lateral buckling deformation is observed in either the internal or external waterproof steel plate.

Elasto-plastic stage (A_1_-B_1_): The total axial compression displacement of PSC-SC increases at an accelerating rate, accompanied by a gradual reduction in compressive stiffness. After reaching the peak load B_1_, lateral deflection begins to develop in the external waterproof steel plate (Fig 7b), whereas the remainder of the specimen remains intact.

Buckling stage (B_1_-C_1_): As the total axial compression deformation continues to increase, the lateral deflection of the external waterproof steel plate markedly enlarges. However, the precast concrete blocks, the inner steel plates, and the internal waterproof steel plates at the joint remain undamaged, with their stress states still in the elastic range.

(2) Axial load-displacement relationship of PSC-CFC

As illustrated in Fig 8a, the failure mode of PSC-CFC is primarily manifested as the crushing of precast concrete blocks, and the stress deformation process can also be divided into three stages: the elastic stage (O_2_-A_2_), the elasto-plastic stage (A_2_-B_2_), and the softening stage (B_2_-C_2_).

**Fig 8:**
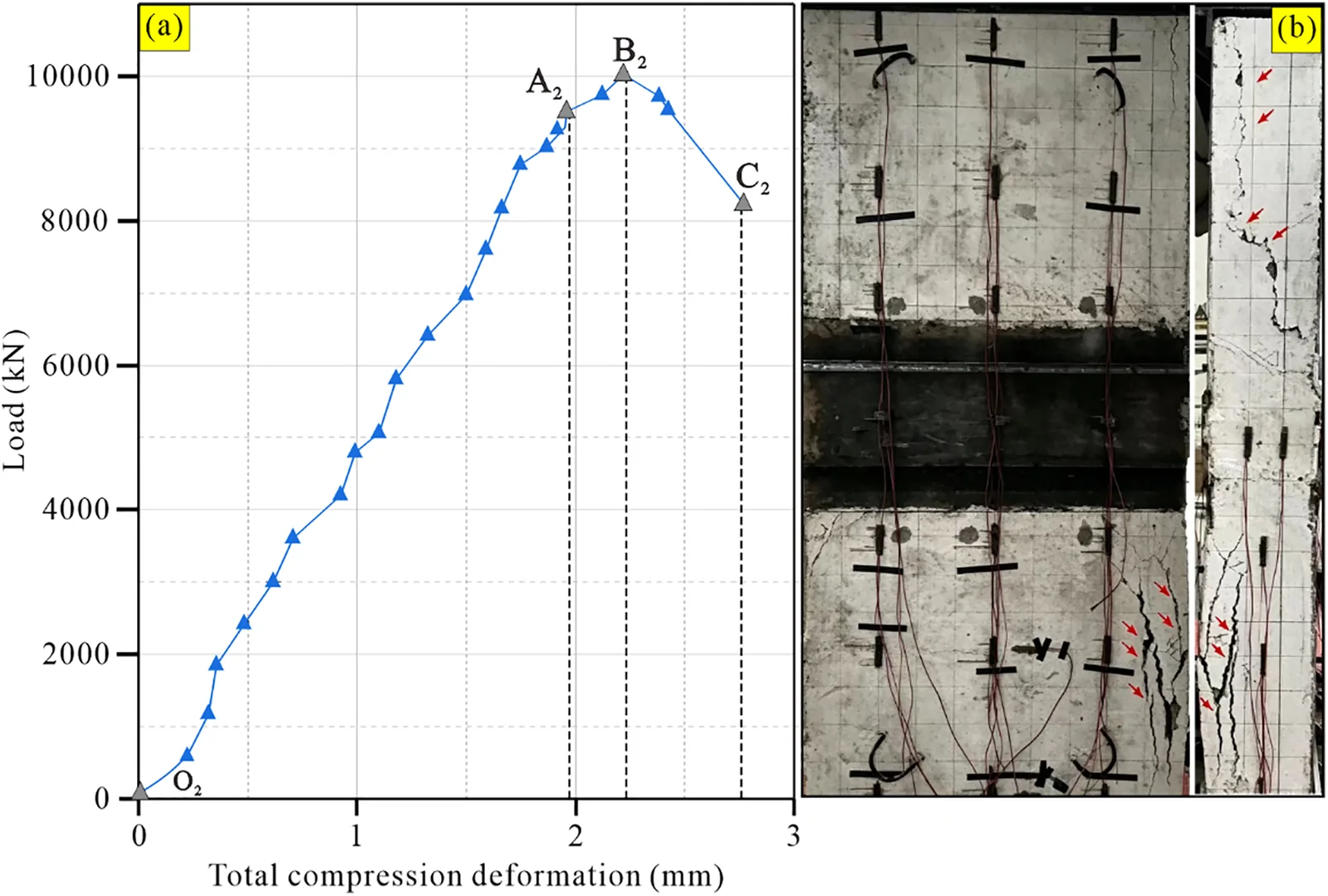
Compressive deformation curve and loading process of PSC-CFC. (a) Load-deformation response under axial compression, showing the relationship between applied load and total compressive deformation with characteristic points (O_1_, A_1_, B_1_, C_1_). (b) Loading and deformation evolution process of the specimen.

Elastic stage (O_2_-A_2_): At this initial stage, no cracks are observed in either the precast concrete blocks or the joint concrete. The connection between the precast concrete and the inner steel plate via shear studs remains intact. The internal and external waterproof steel plates and welds at the joint remain intact, with no signs of lateral buckling deformation observed.

Elasto-plastic stage (A_2_-B_2_): As shown in Fig 8b, cracks gradually develop in the precast concrete blocks. As loading continued, the cracks extended progressively toward both ends, and their widths also gradually increased. Debonding is observed at the ends between the precast concrete block and the inner steel plate. However, at the peak load B_2_ (10004 kN), the internal and external waterproof steel plates, as well as the welds remain intact, and no cracks are observed in the concrete at the joint.

Softening stage (B_2_-C_2_): After the peak load, as the total axial compression deformation continues to increase, both the depth and width of the cracks in the prefabricated concrete blocks increase continuously, while the debonding between the upper prefabricated concrete block and the inner steel plate progressively propagate downward. When the C_2_ point is reached, the specimen cannot continue to carry and stop loading.

### Axial load-strain relationships of PSC-SC and PSC-CFC

(1) Axial load-strain relationship of PSC-SC

Based on the experimentally measured strain data, the axial load-strain curves for PSC-SC are shown in Fig 9. It can be seen that both the concrete-side and steel-side measurement points initially exhibit compressive strains aside from a few outliers. As the load increases, strains on both sides generally increase linearly during the early loading phase. A noticeable inflection point appears in the load-strain curve at an axial load of approximately 6230 kN, where a sudden increase in strain is observed on the steel plate side. At this time, lateral buckling deformation occurs in the external waterproof steel plate at the joint, and the specimen begins to exhibit global arching deformation.

**Fig 9:**
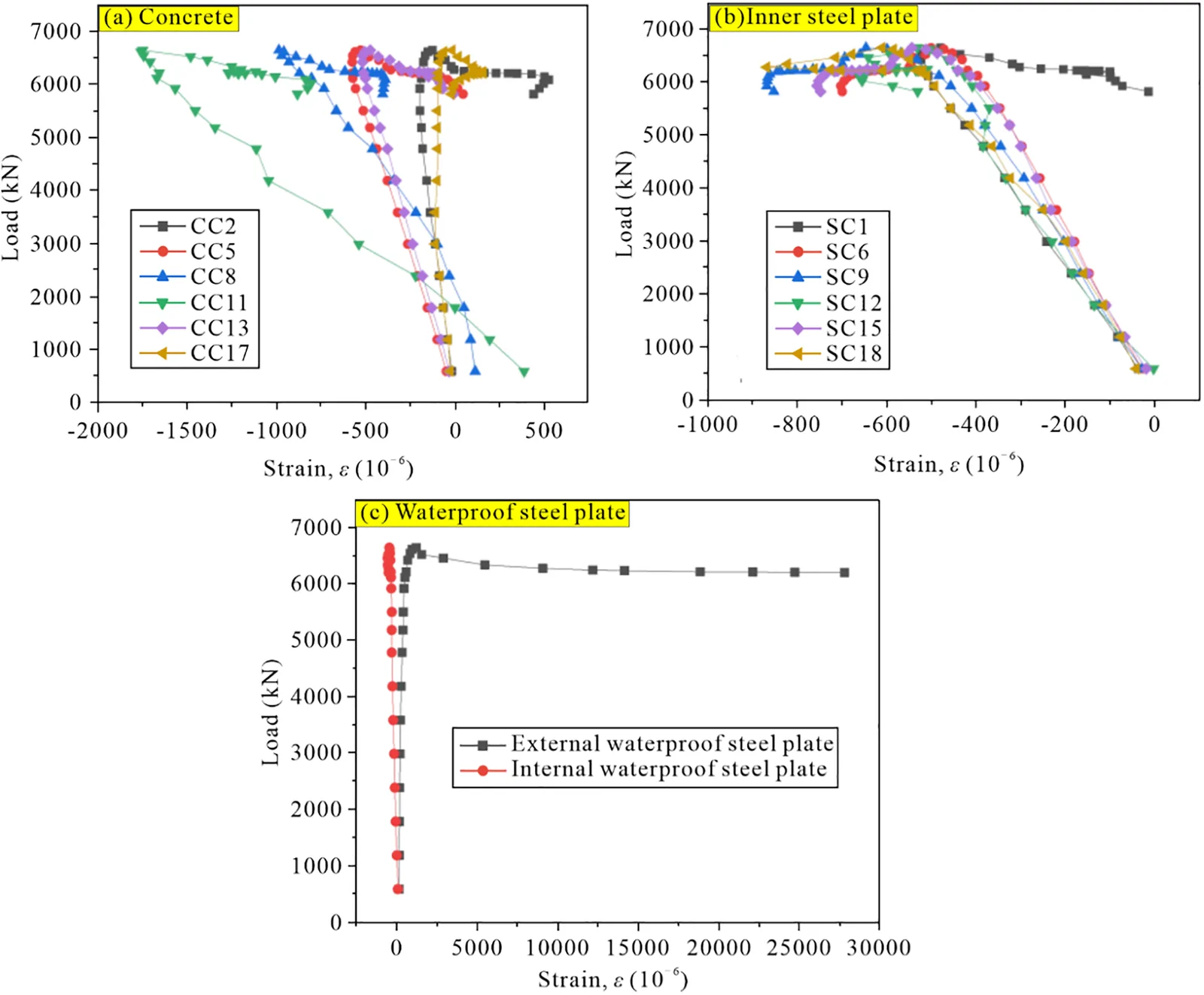
Axial load-strain curves of PSC-SC. (a) Load-strain curves of concrete gauges under axial loading. (b) Load-strain curves of inner steel plate gauges. (c) Load-strain behavior of waterproof steel plates, comparing internal and external steel plate responses under axial compression.

After the load exceeds the peak value, the arching deformation of PSC-SC intensifies further. As shown in Fig 9b, the proportion of the load borne by the steel plate side increases significantly, with its strain rising rapidly in response to the load. The opposite phenomenon observed at measurement point SC1 may be attributed to the influence of the specimen's end. In contrast, it can be seen from Fig 9a that the strain on the concrete side decreases rapidly with increasing load. By the end of loading, tensile strain is observed at measurement points near both ends of the concrete side (CC2, CC5, CC13, and CC17). This phenomenon is mainly due to the pronounced arch-shaped deformation of the specimen. During this process, the limiting angle steels at the top and bottom ends impose lateral constraints on the concrete ends, leading to a tendency for localized tensile stress to develop in these regions.

(2) Axial load-strain relationship of PSC-CFC

For PSC-CFC, it can be seen from Fig 10a that when the axial load reaches 8760 kN, the load-strain curve of the concrete has an inflection point, and the strain increases at an accelerating rate. As shown in Fig 10b, except for the measuring point on the internal waterproof steel plate at the joint, the strain curves at the other locations on the steel plate side also exhibit inflection points. However, the rate of strain increase tends to slow down. In comparison, the strain variation on the external waterproof steel plate is smaller than that on the internal one (Fig 10c). At this time, axial cracks are generated in the prefabricated block near the joint, which reduces the effective compressive area of the concrete, and the upper prefabricated block and the inner steel plate begin to peel off. As the proportion of load carried by the concrete increases, the strain growth rate on the concrete side increases accordingly, while that on the steel-plate side decreases. The internal and external waterproof steel plates, as well as the joint concrete, remain intact and are still in the elastic stage. After peak load, the cracks in the precast block concrete further develop, and the peeling of the upper precast block concrete and the inner steel plate further extend. As a result, the strain on the concrete side continues to increase but the strain on the steel plate side continues to decrease as the load gradually decreases. Owing to the concrete infill in the joint cavity, which significantly enhances the joint stiffness, the load-strain curve of the internal waterproof steel plate remains approximately linear up to the peak load. After the peak load, the strain returns approximately along the original path. The lateral deflection deformation of the external waterproof steel plate is also small.

**Fig 10:**
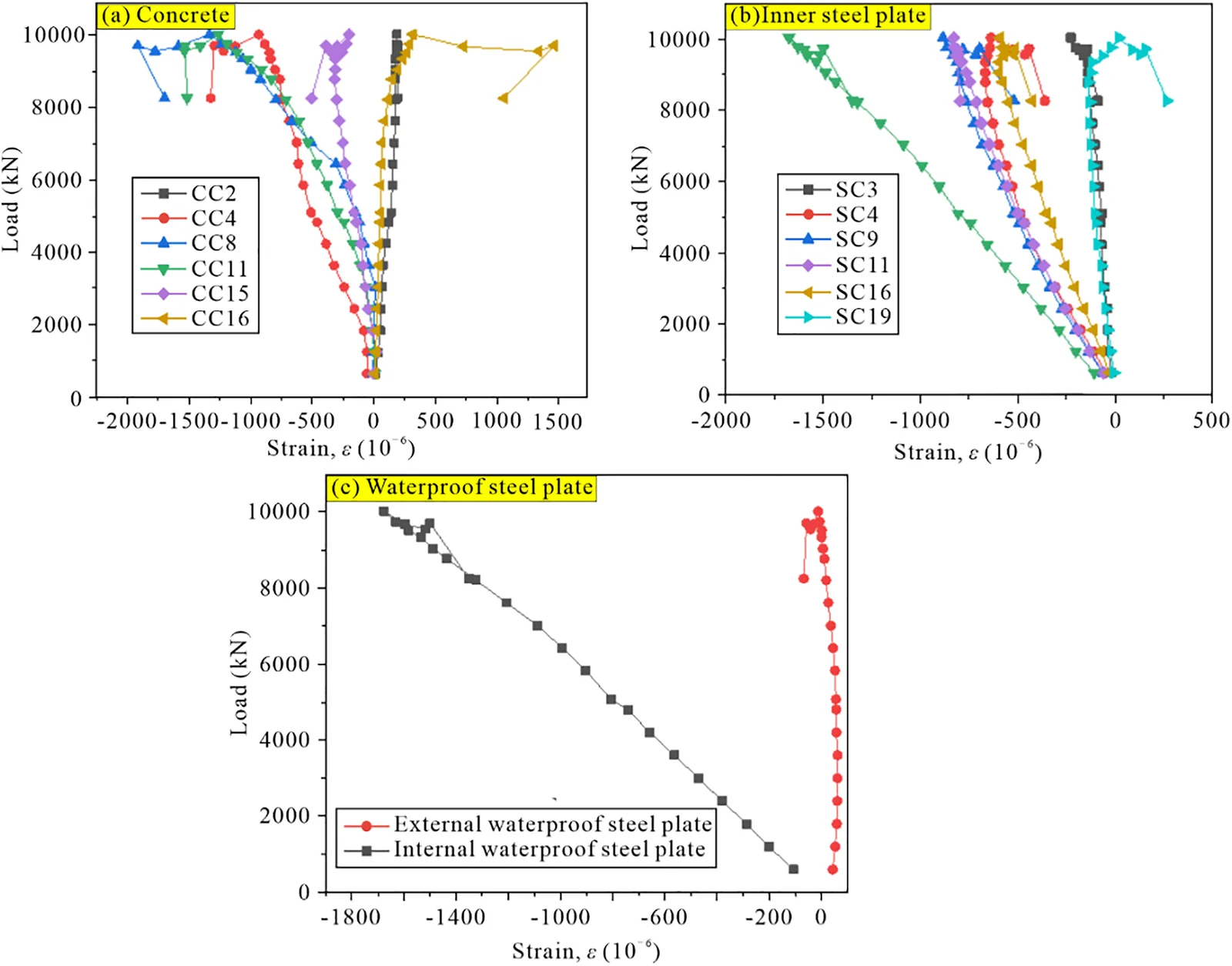
Axial load-strain curves of PSC-CFC. (a) Load-strain curves of concrete gauges under axial loading. (b) Load-strain curves of inner steel plate gauges. (c) Load-strain behavior of waterproof steel plates, comparing internal and external steel plate responses under axial compression.

## Three-dimensional numerical simulation

### Constitutive models of materials

The concrete constitutive model is based on the concrete plastic damage model proposed by Lubliner et al. [42] and subsequently modified by Lee et al. [43] in 1998. The model assumes that the elastic behavior of materials is linearly elastic and isotropic. The inelastic behavior of concrete under uniaxial tension and compression is described by introducing damage factor. The elastic modulus *E*_*c*_ of concrete is calculated by the following formula:


$$\begin{array}{c} E_{c}=\frac{10^{5}}{2.2+34.7/f_{cu}} \end{array}$$ (1)

where, *f*_*cu*_ represents the compressive strength of the concrete cube.

The damage variables are used to describe and quantify the extent and progression of damage in concrete due to tensile cracking and compressive crushing. The calculation method follows the approach proposed by Sidoroff [44]:


$$\begin{array}{c} d=1-\sqrt{\frac{\sigma_{c}}{E_{c}\varepsilon_{c}}} \end{array}$$ (2)

where, *σ*_*c*_ denotes the stress in the concrete, and *ε*_*c*_ represents the strain in the concrete.

In addition, only the cubic specimens cast simultaneously with the test specimens are subjected to compressive tests. The conversion formula between the cubic compressive strength *fcu* and the prism compressive strength *f*c used in the concrete constitutive model is expressed as:


$$\begin{array}{c} f_{c}=0.76f_{cu} \end{array}$$ (3)

The stress-strain relationship of the steel material adopts a bilinear model [45], in which the stress-strain curve is uniquely defined, facilitating numerical convergence during analysis. The material properties of the steel are based on the experimental results (Table 1), with an elastic modulus of *E*_*a*_ = 2.06 × 10^6^ MPa and a poisson’s ratio of 0.3.

### Establishment of finite element model

The finite element model is developed based on the actual dimensions of the specimens. The component models include concrete, steel plates, shear studs, and welds. The finite element models of PSC-SC and PSC-CFC are shown in Fig 11. The C3D8R elements are adopted for all solid components. The PSC-SC and PSC-CFC models consist of approximately 146,000 and 156,000 elements, respectively. The mesh size of the precast concrete block is 25 × 21.4 × 50 mm, while that of the inner steel plate is 25 × 10 × 50 mm. The shear studs are embedded into the concrete using the embedded constraint method, and are rigidly connected to the steel plates. The interaction between the steel plates and concrete is modeled using contact elements. In the tangential direction, a coulomb friction model is adopted with a friction coefficient *μ* = 0.6 [46]. In the normal direction, ‘hard contact’ is used to simulate the contact behavior.

**Fig 11:**
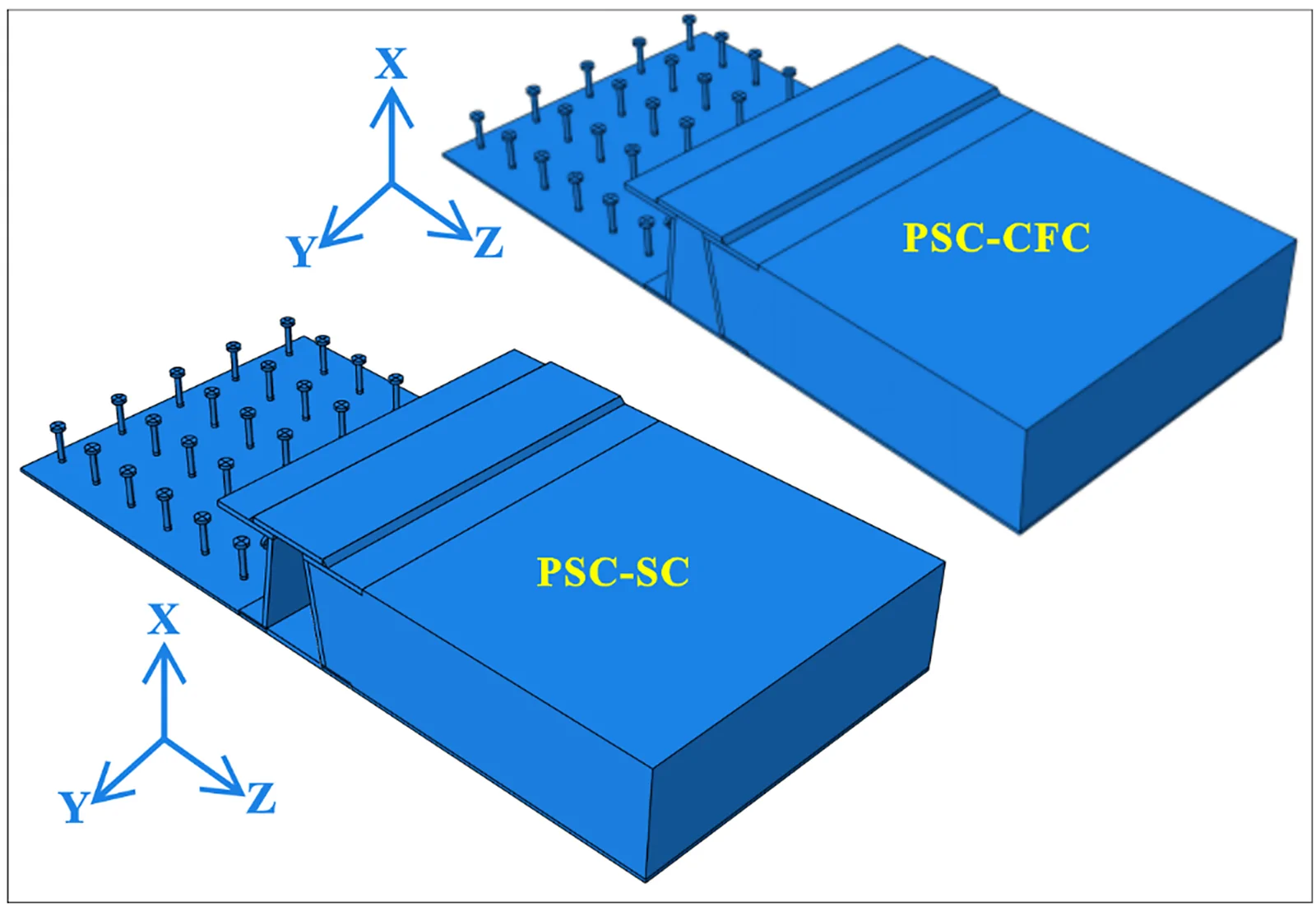
Finite element models of specimens with two different joints.

### Grid division and boundary condition setup

Structured meshing is employed for all structural components to generate regular, low-distortion elements. A uniform mesh size is used for most regions of the model, with local refinement introduced in areas prone to stress concentrations or geometric discontinuities, particularly around the joint. A mesh independence study is conducted using different mesh densities. Under the same axial compressive load, the maximum displacement of the specimen is selected as the evaluation index. When the relative difference in the maximum displacement between two successive mesh densities is less than 1%, the numerical results are considered to be mesh-independent. Based on this criterion, the final mesh configuration adopted in this study is shown in Fig 12. In the model, loading is applied through displacement control on the loading end surface. The boundary condition settings mainly include two aspects. Firstly, on the loading face, the displacement constraints in the X and Y directions and the rotation constraints are applied to all nodes. Secondly, displacement constraints in the X, Y, and Z directions, along with rotational constraints about the X and Y axes, are imposed on all nodes on the fixed end face.

**Fig 12:**
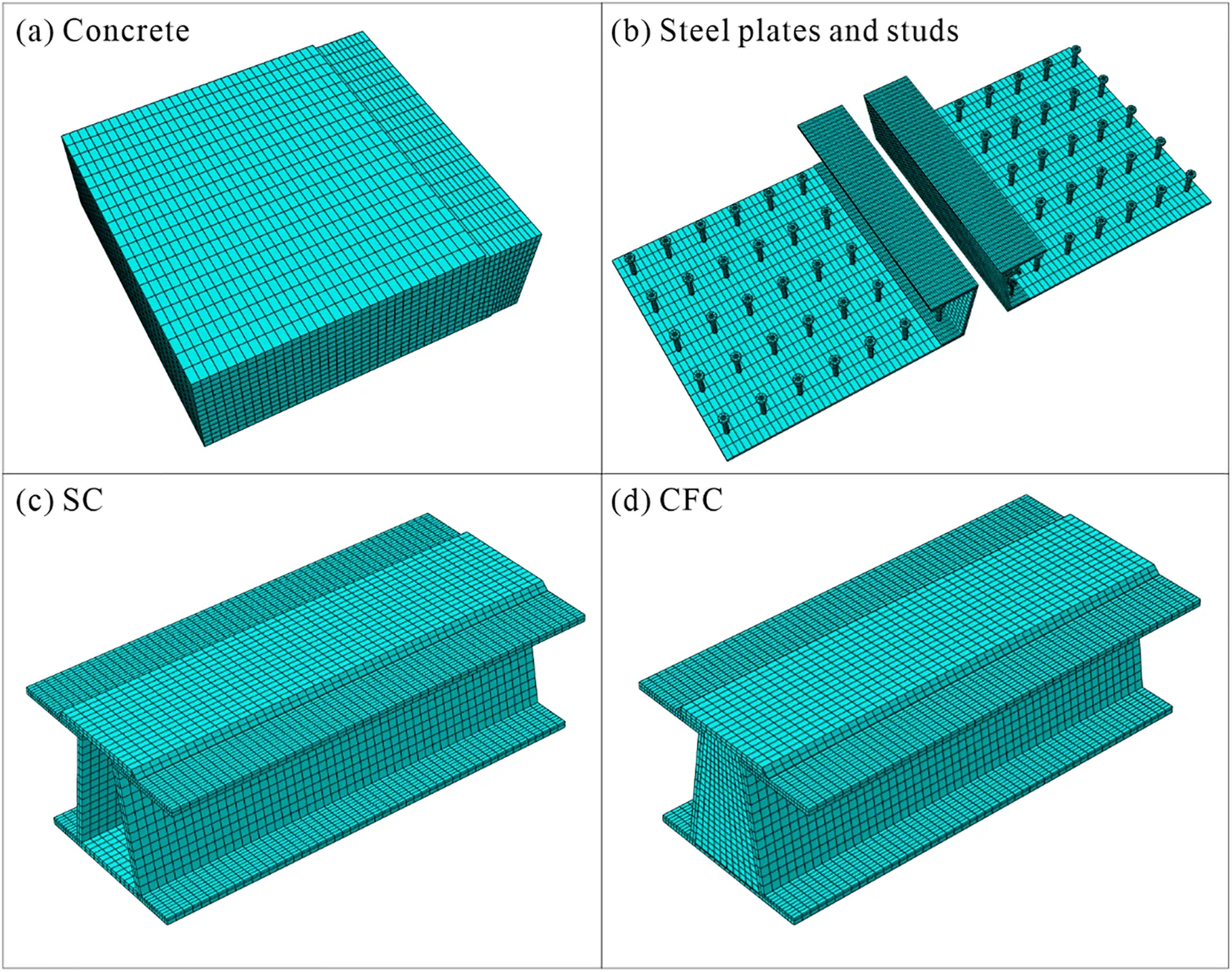
Grid division of different components in SC joint and CFC joint.

### Numerical simulation results and analysis

#### Comparison between numerical simulation and experimental results.

As shown in Fig 13a and Fig 13b, a comparison between the failure modes observed in the numerical simulation and those from the experiment reveals good agreement. In the numerical results, PSC-SC exhibits bending and buckling failure of the external waterproof steel plate at the joint. For the PSC-CFC specimen, the filled concrete enhances the compressive capacity of the joint. As the axial compressive load increases, crushing failure first occurs in the precast concrete block, followed by damage to the joint. In addition, it can be seen from the cloud diagram of the damage factor of the precast block concrete during failure that the part with the larger damage factor is the same as the crack position measured in the test (Fig 13b).

**Fig 13:**
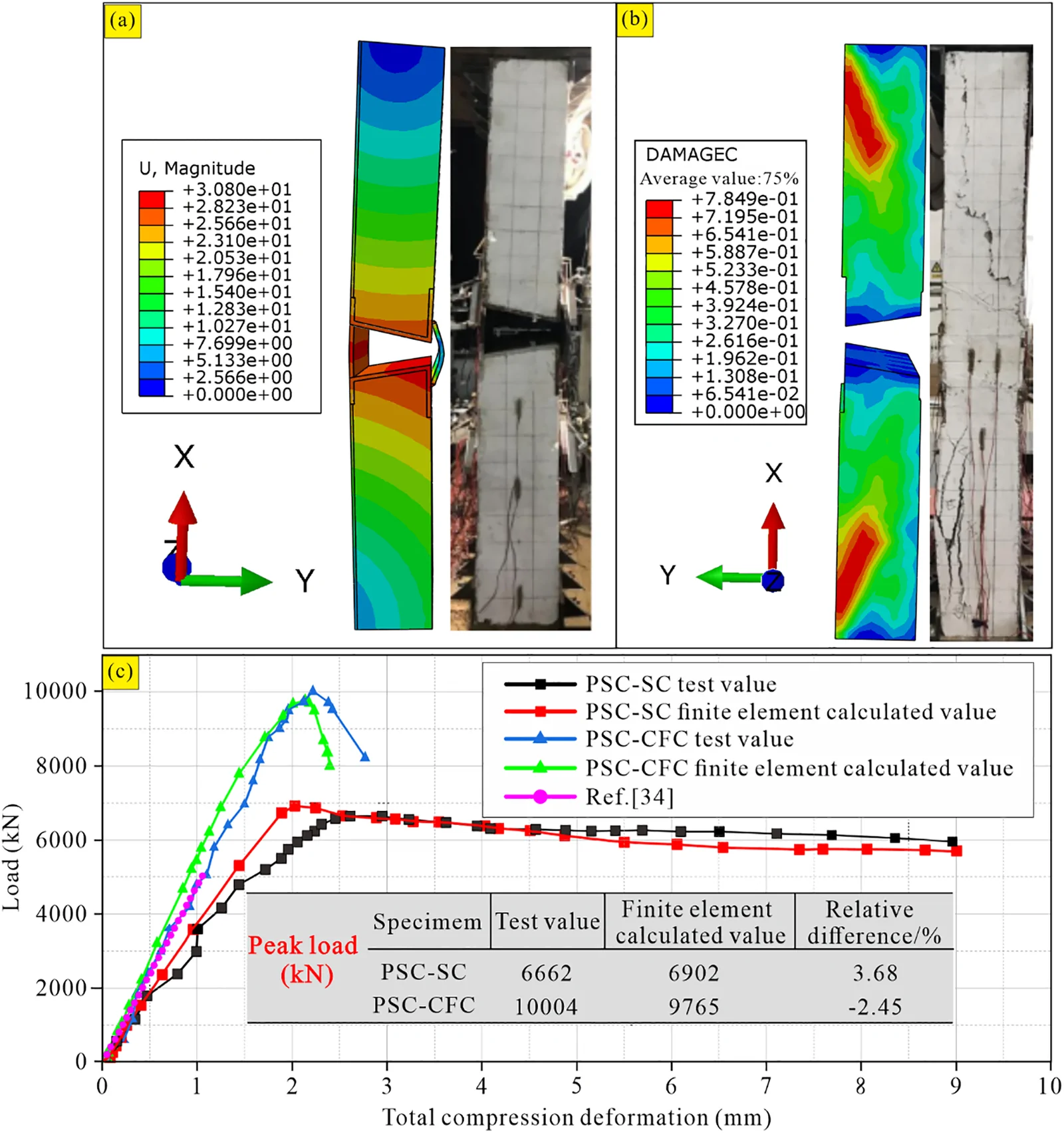
Comparison between numerical simulation results and experimental results. (a) and (b) present comparisons of the failure modes between numerical simulations and experimental observations for the PSC-SC and PSC-CFC specimens, respectively. (c) shows the comparison of load-deformation responses between experimental tests and finite element analyses for both PSC-SC and PSC-CFC specimens, including peak load comparison and relative error evaluation.

As illustrated in Fig 13c, a further comparison of the load-deformation curves of the specimens also shows a good agreement. In particular, the simulated values in the elastic stage show strong agreement with the experimental data. This indicates that the finite element model can accurately capture the initial stiffness of the specimens. After reaching the peak load, the simulated curve shows a more rapid decline in load-bearing capacity compared to the experimental results, which may be attributed to idealized boundary conditions, limitations in measurement equipment, and simplifications in the material model. The relative difference between the simulated and experimental peak loads is only 3.68% for PSC-SC and −2.45% for PSC-CFC. Therefore, it can be concluded that the adopted finite element modeling approach is effective, and the numerical model can reliably reproduce both the experimental process and the mechanical behavior of the specimens.

Furthermore, Fig 13c compares the compressive performance of the two proposed joint configurations with that of the joint reported in Ref [34]. It should be noted that the specimen in Ref [34]. is loaded only up to 5,000 kN. One can see that the three-steel-plate joint proposed in Ref [34]. exhibits an initial compressive stiffness comparable to that of the PSC-CFC specimen. This indicates that welding additional steel plates within the cavity can effectively enhance the compressive stiffness. However, compared with filling the cavity with concrete, incorporating additional welded steel plates increases fabrication and construction complexity.

#### Mechanism analysis of specimen based on numerical simulation.

(1) Mechanical behavior of PSC-SC

The overall load-deformation curve of PSC-SC can be divided into three stages based on the stress development process of steel components. These stages are the elastic stage (O_1_-A_1_), the elastic-plastic stage (A_1_-B_1_), and the buckling stage (B_1_-C_1_). The load-deformation curves of PSC-SC and its components during loading are shown in Fig 14, while the maximum axial stresses of the different components at key cross-sections are presented in Table 3. For each component, only the peak axial stress at the critical location is reported. Based on Fig 14 and Table 3, the stress distribution and load-bearing mechanisms of PSC-SC at each stage are described as follows:

**Table 3:** Maximum stress at the midspan section of the prefabricated block and the PSC-SC.

Key point	Prefabricated block (MPa)		Joint (MPa)	
	Concrete	Inner steel plate	Internal waterproof steel plate	External waterproof steel plate
A_2_	17.77	138.61	268.24	346.76
B_2_	17.59	136.10	284.76	376.32
C_2_	18.51	149.17	379.44	583.53

**Fig 14:**
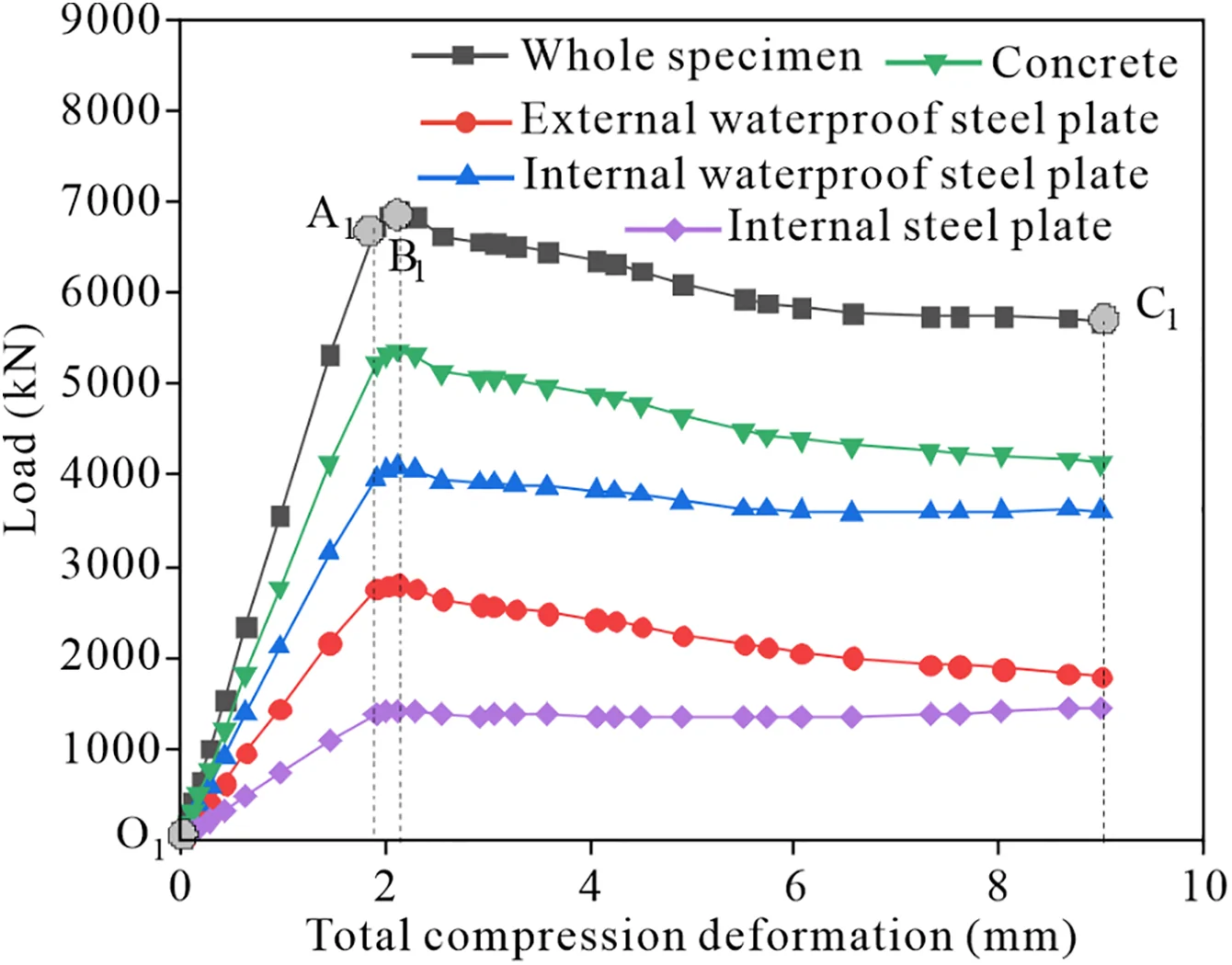
Load-deformation curves of PSC-SC.

Elastic Stage (O_1_-A_1_): During the elastic stage, the load-displacement responses of all load-carrying components are approximately linear. Therefore, the load carried by each component is assumed to be proportional to its axial stiffness. The load-sharing ratio of each component is calculated as the ratio of its axial stiffness (i.e., the slope of its load-displacement curve) to the sum of the axial stiffnesses of all load-carrying components. Based on this method, the inner steel plate and the concrete of the prefabricated block carry 20.3% and 79.7% of the load, respectively. At the joint region, the internal waterproof steel plate bears 59.1% of the load, while the external waterproof steel plate carries 40.9%. The load borne by the inner waterproof steel plate is approximately 1.44 times that carried by the outer waterproof steel plate. This phenomenon is attributed to the fact that the prefabricated silo wall block is composed of a single-sided steel plate and the neutral axis located closer to the steel side. As a result, the internal waterproof steel plate, being nearer to the neutral axis, bears a larger portion of the load. Overall, in PSC-SC, the majority of the load (about 80%) is carried by the concrete in the prefabricated block. The load distribution between the internal and external waterproof steel plates at the joint is uneven, with the inner waterproof steel plate bearing a greater share. At the A_1_ load level, the maximum axial compressive stresses in the concrete and steel plate at the mid-span of the prefabricated block are 17.77 MPa and 138.61 MPa, respectively. both well below their material limits (31.92 MPa for concrete and 330 MPa for the steel plate). It should be noted that although the internal waterproof steel plate carries a larger axial force than the external one, the latter experiences not only axial compression but also significant bending deformation due to eccentricity. Under the combined effect of these deformations, the stress in the external waterproof steel plate becomes even higher. At the mid-span cross-section, the maximum stresses in the internal and external waterproof steel plates reach 268.24 MPa and 346.76 MPa, respectively. The stress in the external waterproof steel plate exceeds its yield strength (343 MPa), which indicates the onset of local yielding in this region and marking it as the first part of the structure to enter plastic deformation.

Elastoplastic stage (A_1_-B_1_): During this stage, the inner and outer waterproof steel plates at the joint carry the majority of the axial load, and their axial compressive stresses increase rapidly with the applied load. In contrast, the axial stresses in the steel plate and concrete of the prefabricated block change only marginally, indicating that the applied load is primarily transferred through the joint steel plates. As the load continues to increase, the outer waterproof steel plate at the joint exhibits pronounced out-of-plane local buckling at the peak load (Point B_1_) due to eccentric compression. At this stage, the outer steel plate sustains a maximum axial compressive stress of 376.32 MPa, while the tensile side is subjected to a relatively high axial tensile stress. In comparison, the inner waterproof steel plate exhibits no local buckling and reaches a maximum axial stress of 284.76 MPa. This result demonstrates its stable load-carrying behavior. According to the local stability theory of steel plates, the onset of local buckling leads to a rapid reduction in the effective compression area, accompanied by a significant degradation in axial stiffness and load-carrying capacity, which in turn induces stress redistribution within the joint and prevents the specimen from sustaining additional external load. Therefore, the ultimate bearing capacity of the PSC-SC specimen is primarily governed by the local buckling instability of the outer waterproof steel plate.

Yield stage (B_1_-C_1_): The load-deformation curve of the PSC-SC enters the descending section. During this stage, the lateral buckling deformation of the external waterproof steel plate at the joint further develops. Due to the large axial strain, its load-bearing capacity decreases rapidly. Meanwhile, as the load on the internal waterproof steel plate at the joint gradually increases, its axial stress rises quickly. At point C_1_, the maximum axial stress in the external waterproof steel plate at the joint mid-span reaches 583.53 MPa, while that of the internal waterproof steel plate is 379.44 MPa, which indicates that both internal and external waterproof steel plates have yielded.

(2) Mechanical behavior of PSC-CFC

Combined with the load-deformation curve of PSC-CFC (Fig 15) and the maximum axial load value (Table 4) at the key points of the specimen at different positions, the stress mechanism of each stage is analyzed as follows.

**Table 4:** Maximum stress at the midspan section of the prefabricated block and the PSC-CFC.

Key point	Prefabricated block (MPa)		Joint (MPa)		
	Concrete	Inner steel plate	Internal waterproof steel plate	Joint concrete	External waterproof steel plate
A_2_	27.22	170.46	192.02	24.84	57.26
B_2_	33.37	196.73	210.80	26.73	61.21
C_2_	40.57	95.53	144.04	16.91	50.17

**Fig 15:**
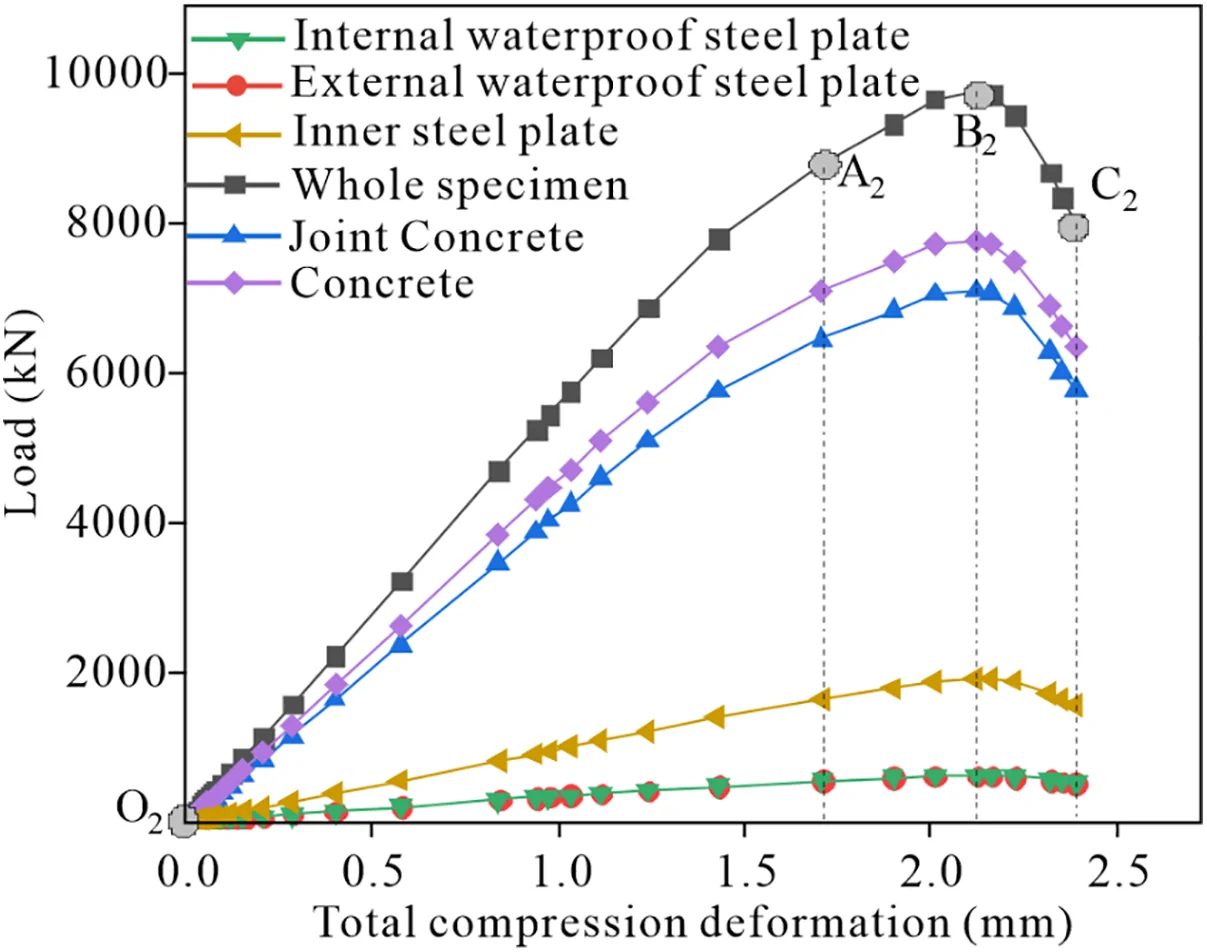
Load-deformation curves of PSC-CFC.

Elasto-stage (O_2_-A_2_): the inner layer steel plate and outside concrete of PSC-CFC bear 18.7% and 81.3% of the load, respectively. At the same time, the loads borne by the internal waterproof steel plate, concrete and external waterproof steel plate at the joint are 13.0%, 74.0% and 13.0%, respectively. Compared with SC, after filling the joint cavity with concrete, the internal and external waterproof steel plates bear the same load, and the load-displacement curves throughout the entire process show good agreement. This fully shows that the presence of joint concrete reduces the influence of the neutral axis, making precast block concrete and joint concrete the main components bearing the load. At the load level corresponding to point A_2_, the maximum axial compressive stresses in the mid-span section of the prefabricated block concrete and steel plate are 27.22 MPa and 170.46 MPa, respectively. For the mid-span section of the joint, the maximum axial compressive stresses in the internal and external waterproof steel plates are 192.02 MPa and 57.26 MPa, respectively, while the stress in the joint concrete reaches 24.84 MPa (with a measured compressive strength of 18.24 MPa). Therefore, during this stage, only the local stress in the joint concrete slightly exceeds its prismatic compressive strength. However, the influence of this localized overstress is minimal, the load-displacement curves of the joint concrete remain linear in the O_2_-A_2_ segment.

Elastoplastic stage (A_2_-B_2_): During this stage, the axial compressive stresses in the PSC-CFC specimen and its constituent components increase continuously with the applied load. The load-displacement responses of the inner steel plate in the prefabricated block and the waterproof steel plate at the joint remain approximately linear, indicating that neither steel plate has experienced significant yielding and that sufficient load-carrying capacity is still available. In contrast, the axial compressive stress in the concrete of the prefabricated block increases most significantly, reaching 33.37 MPa at the peak load (Point B_2_), which is higher than the measured prism compressive strength of 31.92 MPa. This indicates that the concrete in the prefabricated block is the first to reach its compressive strength limit and undergo crushing. According to the compressive behavior of concrete, the onset of concrete crushing is accompanied by a pronounced reduction in stiffness, which promotes stress redistribution within the specimen and consequently diminishes the concrete’s capacity to effectively sustain and transfer axial compressive loads. Consequently, the overall load-carrying capacity of the specimen decreases rapidly after the peak load. Beyond Point B_2_, the load-carrying capacities of both the prefabricated-block concrete and the joint concrete deteriorate simultaneously, indicating that the ultimate bearing capacity of the PSC-CFC specimen is primarily governed by the compressive failure of these two concrete regions. Since the steel plates remain below their yielding limit, their primary role is to provide confinement to the surrounding concrete and delay structural instability, rather than to govern the ultimate load-carrying capacity.

Softening stage (B_2_-C_2_): The load-displacement curves of PSC-CFC and its components all enter the descending stage. Except for the axial compressive stress in the prefabricated block concrete, which continues to increase, the axial compressive stresses in the other components decrease. At point C_2_, the maximum compressive stress in the prefabricated block concrete reaches 40.57 MPa. This is due to the loss of load-bearing capacity in some concrete sections caused by plastic damage, which reduces the effective compression area of the concrete. As a result, the axial compressive stress in the prefabricated block concrete continues to increase even as the applied load decreases.

## Effects of joint design parameters on the compressive performance of the silo wall

### Joint parameters of PSC-SC

For the PSC-SC, both the experimental results and finite element analysis indicate that the failure is caused by large lateral buckling and instability of the external waterproof steel plate. Therefore, the joint design parameters for PSC-SC primarily focus on the effects of the connection length *l* and thickness *d* of the external waterproof steel plate. Table 5 presents the selected values of the joint design parameters and the corresponding key results. Using the finite element model PSC-SC-*l*240 as the baseline model, each comparative model modifies a single parameter while keeping all other parameters constant. The resulting load-deformation curves for the various finite element models are shown in Fig 16.

**Table 5:** Design parameters and main numerical simulation results for PSC-SC.

Finite element model	l (mm)	d (mm)	Elastic stage displacement (mm)	Elastic stage stiffness (kN/mm)	Peak load (kN)
PSC-SC-l160	160	16	1.90	3739.6	7470.0
PSC-SC-l200	200	16	1.90	3722.0	6980.0
PSC-SC-l240	240	16	1.90	3694.4	6900.0
PSC-SC-d10	240	10	0.96	3687.5	4510.0
PSC-SC-d20	240	20	1.90	3708.3	7550.0

Note: PSC-SC represents the finite element model with SC joint. *l* represents the connection length of the external waterproof steel plate. *d* represents the thickness of the external waterproof steel plate.

**Fig 16:**
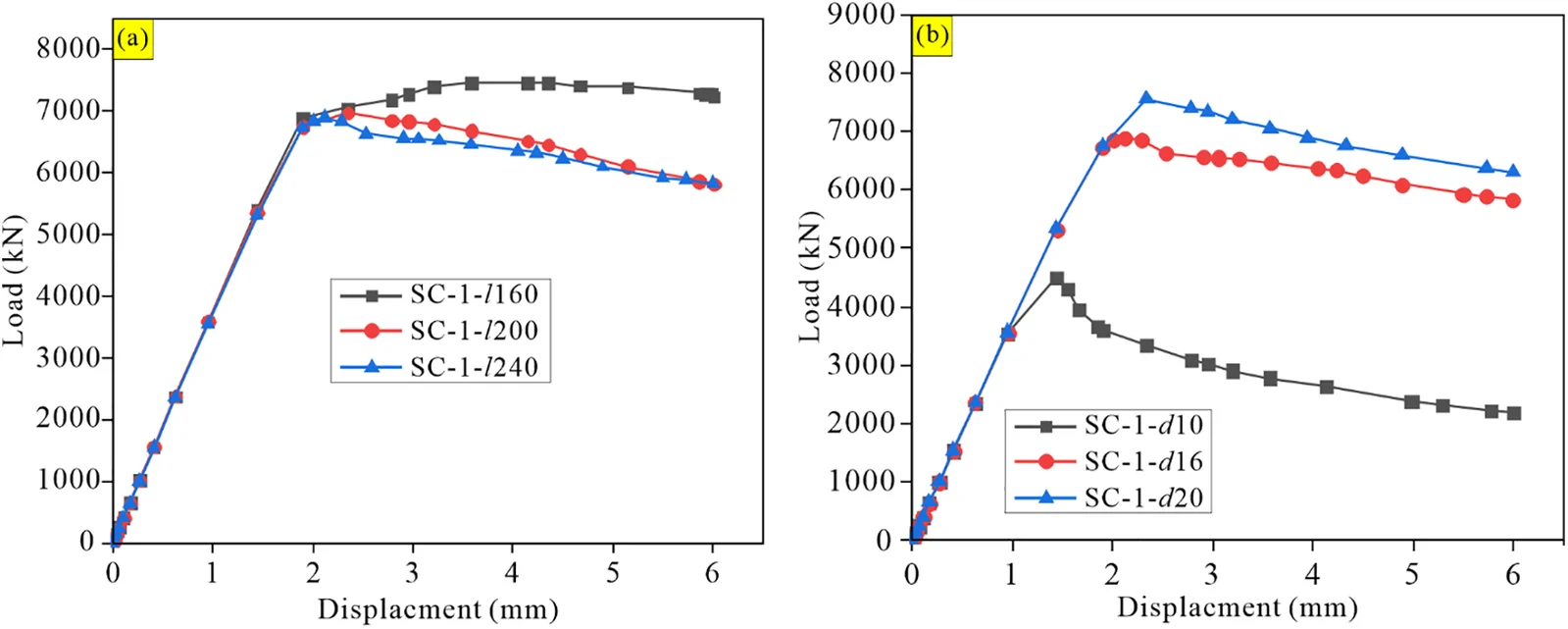
Load-displacement curves of PSC-SC with different design parameters. (a) Effect of the connection length of the external waterproof steel plate on the load-displacement behavior of PSC-SC specimens. (b) Effect of the thickness of the external waterproof steel plate on the load-displacement behavior of PSC-SC specimens.

(1) Connection length of external waterproof steel plate

As shown in Fig 16a, with the increase in the connection length of the external waterproof steel plate, the displacement in the elastic stage and the initial stiffness of the finite element model remain nearly unchanged, whereas the peak load exhibits a gradual reduction. When the connection length increases from 160 mm to 240 mm, the elastic stage displacement remains constant at 1.90 mm, and the peak load obtained from the finite element analysis decreases by 6.56% and 1.15%, respectively. Moreover, the shorter the connection length of the external waterproof steel plate, the more fully the specimen develops plasticity. The displacements in the elastic-plastic stage for connection lengths of 160 mm, 200 mm, and 240 mm are 2.24 mm, 0.44 mm, and 0.22 mm, respectively. It can be seen that the connection length of the external waterproof steel plate has little influence on the elastic stage of the specimen but significantly affects its plastic development and peak load.

(2) Thickness of external waterproof steel plate

As shown in Fig 16b, an increase in the thickness of the external waterproof steel plate has a negligible effect on the initial stiffness of finite element models. The displacement in the elastic stage increases significantly when the thickness increases from 10 mm to 16 mm, while no change is observed between 16 mm and 20 mm thickness. This indicates that once the thickness of the external water-stop steel plate reaches a certain level, further increases have minimal impact on both the elastic stage displacement and the initial stiffness. As the thickness increases from 10 mm to 16 mm and then to 20 mm, the peak load obtained from the finite element analysis increases by 53.00% and 9.42%, respectively. Therefore, when designing the silo wall joints, the thickness of the external waterproof steel plate should neither be too small nor too large. If the thickness is too small, it significantly reduces the peak load-bearing capacity of the silo wall specimen. Conversely, an excessively large thickness offers only limited improvement in the elastic-stage displacement and peak load, resulting in unnecessary material consumption. Therefore, the steel plate thickness should be reasonably selected to balance structural performance and cost-effectiveness.

### Joint parameters of PSC-CFC

Due to the filling of concrete in the joint cavity, the compressive strength of the CFC joint of the specimen is significantly improved, and the crushing failure of the precast block concrete occurs before the joint. Therefore, the design parameters of PSC-CFC can be regarded as the strength of the concrete at the joint. To investigate the compressive bearing capacity of the joint, the materials of the prefabricated blocks in the finite element model are assumed to be linear elastic. The outside bound steel plate, internal and external waterproof steel plates, and joint concrete are modeled as elastic-plastic materials. Table 6 lists the design parameter values and main results. The load-deformation curves obtained from the finite element model are shown in Fig 17.

**Table 6:** Design parameters and main numerical simulation results for PSC-CFC.

Finite element model	Concrete strength (MPa)	Elastic stage displacement (mm)	Elastic stage slope (mm)	Peak load (kN)
PSC-CFC-C30	30	3.44	4978.95	47800
PSC-CFC-C40	40	3.60	5015.79	49600
PSC-CFC-C50	50	3.93	5021.46	51400

Note: PSC-CFC represents the finite element model with CFC joint. C represents the concrete strength at the joint.

**Fig 17:**
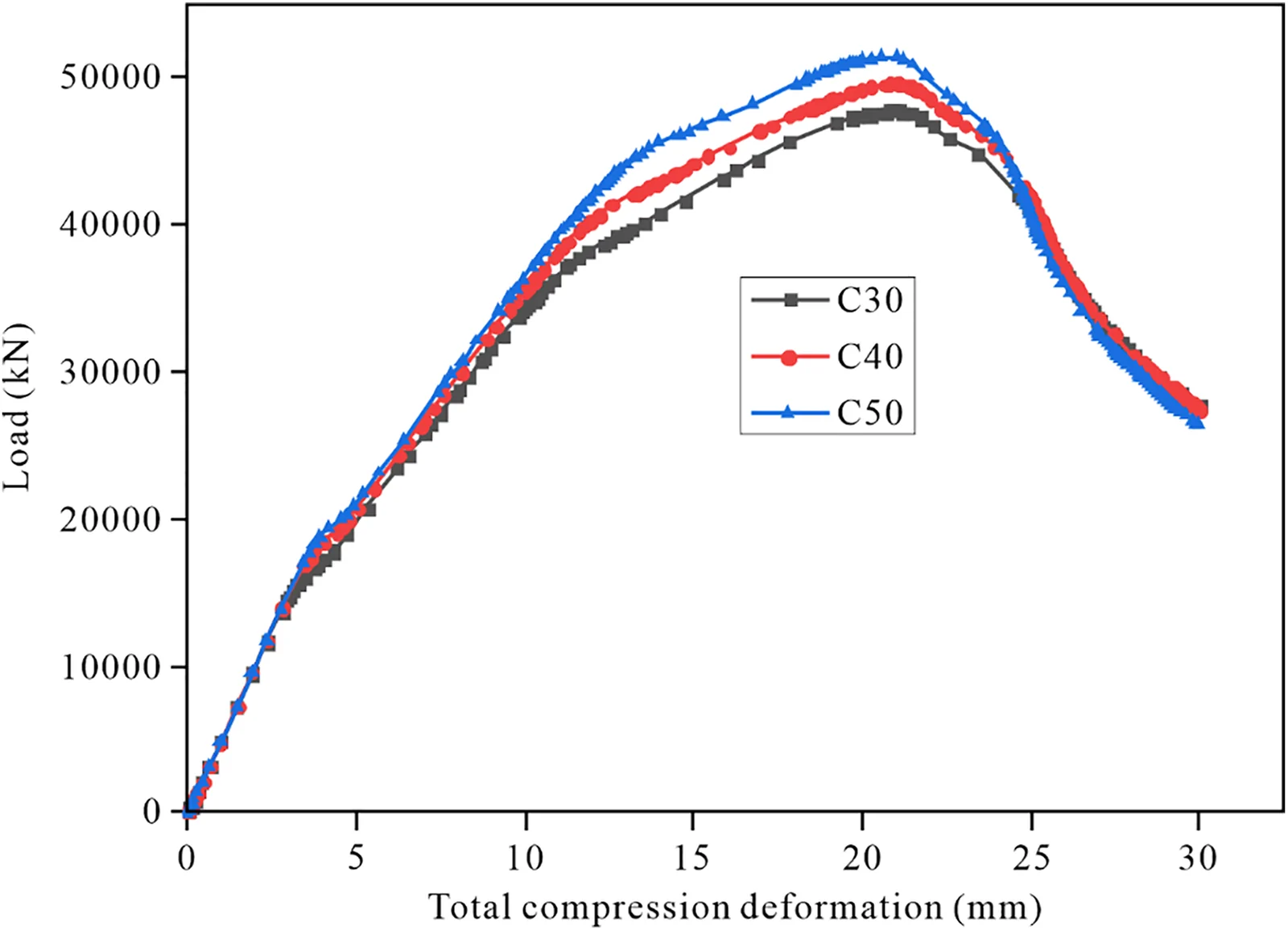
Load-deformation curves of PSC-CFC under different concrete strengths.

As shown in Fig 17, with the increase in joint concrete strength, the elastic-stage displacement, initial stiffness, and peak load of the finite element model all exhibit corresponding improvements. As the joint concrete strength increases from 30 MPa to 40 MPa and then to 50 MPa, the peak load obtained from the finite element analysis increases by only 3.77% and 3.63%, respectively, indicating that the influence of concrete strength on the compressive capacity becomes relatively limited within the investigated strength range. However, compared with the compressive load-carrying capacity of the SC joint, that of the CFC joint increases by more than seven times. Among the investigated design modifications, concrete infilling of the joint cavity is the most effective approach for enhancing the compressive capacity of the joint, compared with increasing the waterproof steel plate thickness or reducing its connection length. Therefore, for the proposed CFC joint under axial compression and within the investigated concrete strength range (30–50 MPa), the use of excessively high-strength infill concrete is not necessary, since only marginal improvements in the peak load are achieved.

## Discussion and analysis

Based on experimental results and numerical simulations, a comparison of the compressive performance of PSC-SC and PSC-CFC under different parameters is presented in Table 7. As shown in Table 7, the two types of joint specimens exhibit marked differences in terms of initial stiffness, yield load, peak load, and post-yield plastic development capacity, which are primarily attributed to the differences in their joint configurations. A detailed analysis is provided as follows:

**Table 7:** Comparison of compressive performance of specimens under different parameters.

Specimens	Parameters					
	*l* (mm)	*d* (mm)	Concrete strength (MPa)	Initial stiffness (kN/mm)	Yield load (kN)	Peak load (kN)
PSC-SC	160	16	25	3740	6853	7470
	200	16	25	3722	6711	6980
	240	16	25	3694	6700	6902
	240	10	25	3688	3529	4510
	240	20	25	3708	6689	7550
PSC-CFC	^*^240	^*^16	^*^25	^*^4907	^*^7722	^*^9765
	240	16	30	4979	13526	47800
	240	16	40	5016	13746	49600
	240	16	50	5021	13671	51400

Note: For PSC-CFC, the “*” indicates that the final failure mode is the crushing of the prefabricated concrete block, while the absence of the “*” denotes failure occurring at the joint.

Under different parameter conditions, the initial stiffness of PSC-SC varies in a small range, with a minimum value of 3688 kN/mm and a maximum value of only 3740 kN/mm. The initial stiffness of PSC-CFC is substantially higher, reaching a maximum value of 5021 kN/mm. Although both of them show a good linear load-deformation relationship in the elastic stage, PSC-CFC exhibits a higher initial stiffness than PSC-SC, indicating that it has stronger deformation resistance in the early stage of loading. The observed difference can primarily be attributed to the distinct configurations of two joints. The small thickness of the internal and external waterproof steel plates of PSC-SC limits its overall stiffness and makes it difficult to effectively resist deformation in the initial stage. The PSC-CFC fills the joint cavity with concrete to construct a steel-concrete synergistic stress system, in which the concrete effectively bears part of the load originally borne by the waterproof steel plate. This not only improves the bearing capacity of the joint area, but also significantly delays the appearance of buckling and plastic deformation, thus greatly enhancing the initial stiffness of PSC-CFC.

In terms of yield performance, the maximum yield load of PSC-SC is only 6711 kN and the minimum value is only 3529 kN. For PSC-CFC, the yield load reaches 7722 kN even when only the concrete precast block experiences crushing failure. When failure occurs in the joint region, the peak load increases further to 13,746 kN. It can be seen that the yield performance of PSC-CFC is much higher than that of PSC-SC. This is because the waterproof steel plate at the joint of the specimen PSC-SC is prone to local lateral deflection under the action of large axial pressure, resulting in the structure yielding in advance. For PSC-CFC, the concrete at the joint effectively inhibits the local buckling of the steel plate, and at the same time bears the load with the steel plate, delaying the arrival of the yield point and significantly improving the yield bearing capacity of the specimen.

The maximum peak load of PSC-SC is only 7550kN under different parameters. In contrast, the peak load of PSC-CFC reaches 9765 kN when the prefabricated concrete block fails, which far exceeds that of PSC-SC. Under different parameter conditions, the maximum peak load reaches 51,400 kN when failure occurs in the joint region, which is nearly seven times that of the PSC-SC. Although the load-deformation curve of PSC-CFC exhibits certain brittle failure characteristics after the peak load, its ductility can be further enhanced by incorporating fibers into the concrete or by using ductile concrete materials.

In summary, the concrete-filled cavity joint exhibits superior compressive performance compared with the steel cavity joint. Moreover, even the use of lower-strength concrete can substantially improve the compressive capacity of specimens, demonstrating the potential of this joint configuration for practical engineering applications.

## Technical limitations and future research

Although the present study provides full-scale experimental and numerical insights into the axial compressive behavior of the proposed PSC-SC and PSC-CFC joints, the findings should be interpreted within the defined scope of the investigation. The specimens and numerical models are specifically developed to examine monotonic axial compression, which is a representative loading condition for the vertical joints considered in the underground silo wall system. Accordingly, the present conclusions primarily concern the load-transfer mechanisms, failure modes, stiffness, and compressive capacity of the two investigated joint configurations, rather than their behavior under all possible structural and environmental conditions.

Several aspects beyond this scope warrant further investigation. In practical applications, joint performance may be affected by tension, shear, bending, cyclic or combined loading, as well as by construction tolerances, assembly imperfections, material variability, and long-term effects such as creep, shrinkage, fatigue, corrosion, and environmental degradation. Dynamic actions, including seismic and impact loading, may additionally introduce rate- and frequency-dependent responses that are not captured by the present quasi-static tests. In tunnel and underground engineering, these dynamic effects should be considered together with the interaction between the structural system and its surrounding ground, because transient loading may alter load-transfer mechanisms and local stress concentrations at joints. Existing studies on assembled tunnel joints and prefabricated underground structures have demonstrated the engineering relevance of impact and seismic actions to the assessment of underground structural performance [47,48]. Therefore, the applicability of the present quasi-static axial-compression results to dynamic service conditions should be treated with caution. Moreover, the current study considers two joint configurations and focuses on structural performance; therefore, the generalizability of the findings to other joint geometries, field conditions, and lifecycle economic considerations should be further verified. These issues are not treated as independent variables in the present study because their systematic evaluation would require dedicated experimental programs, long-term monitoring, uncertainty and sensitivity analyses, and project-specific economic information. Nevertheless, explicitly recognizing these limitations defines the applicability of the present findings and provides a clear basis for future research on the proposed joint system.

From an engineering geology perspective, extending the proposed joint configurations from controlled laboratory conditions to actual underground projects also requires consideration of the spatial variability and uncertainty of subsurface geomaterials and geological interfaces. The spatial distribution of bedrock and subsurface materials is inherently heterogeneous, while available borehole and geotechnical investigation data are often spatially sparse. Previous geoengineering studies have shown that such heterogeneity and limited subsurface information can introduce considerable uncertainty into three-dimensional geological characterization and influence the reliability of subsequent underground engineering applications [49, 50]. In particular, three-dimensional representations of subsurface conditions and explicit uncertainty quantification can provide useful information for engineering decision-making in underground projects. These site-specific geological uncertainties are not explicitly represented in the present laboratory-based investigation, which focuses on the intrinsic mechanical behavior of the proposed joints under controlled axial compression. Accordingly, the present results should not be interpreted as accounting for ground-condition uncertainty or site-specific geomaterial distributions. Future field-oriented studies should integrate geological characterization, spatial variability of geomaterials, and structural response to further evaluate the applicability of the proposed joint system in actual underground environments.

For future field monitoring and data-driven assessment, the temporal dependency and heterogeneity of sequential engineering data also warrant consideration. Measurements collected over successive construction or monitoring stages may be affected by changes in operating conditions, sensor response, environmental disturbances, and other site-specific factors, which can lead to outliers, inconsistent data distributions, and reduced comparability across datasets. Recent work on large-scale sensor-based measurement-while-drilling data has demonstrated the practical importance of dynamic data filtering, normalization, and continuous database updating for processing newly acquired data and establishing a unified database [51]. Such procedures are particularly relevant when engineering data are collected sequentially under different operational and subsurface conditions, where variations in drilling conditions, equipment, subsurface heterogeneity, vibration and shock, and data transmission may affect data quality and comparability. These findings provide useful guidance for future field-oriented monitoring of underground structures, where dynamic filtering and consistent normalization across different operating conditions may be required to establish reliable and comparable structural-response datasets. However, such sequential data-processing and database-updating procedures are beyond the scope of the present laboratory investigation and are therefore identified as an important direction for future research.

## Conclusions

(1) The PSC-SC specimen exhibited three typical stages during axial compression: elastic, elastic-plastic, and yielding stages. The failure was mainly attributed to the lateral deformation and instability of the external waterproof steel plate at the joint. The load-bearing capacity of this joint is therefore mainly governed by the stability performance of the steel plate.
(2) The PSC-CFC specimen experienced elastic, elastic-plastic, and softening stages under axial loading. The infilled concrete effectively improves the load-transfer mechanism within the joint and reduces the risk of steel plate instability. Compared with PSC-SC, the concrete-filled cavity joint exhibits enhanced initial stiffness and a more reliable load-transfer path.
(3) Numerical results demonstrate that the load carried by different components varies significantly between the two joint types. For PSC-SC, the waterproof steel plates dominate the load transfer during the elastic stage, whereas for PSC-CFC, the infilled concrete becomes the primary load-bearing component.
(4) Parameter analysis shows that increasing the thickness of the waterproof steel plate and reducing the connection length can improve the deformation capacity and ultimate load of PSC-SC. For the proposed PSC-CFC joint under axial compression and within the investigated concrete strength range (30–50 MPa), increasing the strength grade of the infilled concrete has a limited effect on the overall compressive performance.
(5) From an engineering application perspective, the selection of joint types should be based on the specific structural requirements of underground silos. The PSC-SC joint can be considered for projects where construction simplicity and installation efficiency are prioritized, particularly under relatively moderate load demands. In contrast, the PSC-CFC joint is recommended for large-capacity underground silos or structures subjected to higher axial loads, where higher stiffness and enhanced load-carrying capacity are required.

## Supporting information

S1 Data Minimal dataset underlying all figures reported in this study. (XLSX)

S2 Data Minimal dataset underlying all tables reported in this study. (XLSX)
